# Evolutionary ecology of Chagas disease; what do we know and what do we need?

**DOI:** 10.1111/eva.12582

**Published:** 2017-12-25

**Authors:** Alheli Flores‐Ferrer, Olivier Marcou, Etienne Waleckx, Eric Dumonteil, Sébastien Gourbière

**Affiliations:** ^1^ UMR 228, ESPACE‐DEV‐IMAGES, ‘Institut de Modélisation et d'Analyses en Géo‐Environnement et Santé’ Université de Perpignan Via Domitia Perpignan France; ^2^ UMR 5096 ‘Laboratoire Génome et Développement des Plantes’ Université de Perpignan Via Domitia Perpignan France; ^3^ Laboratorio de Parasitología, Centro de Investigaciones Regionales “Dr. Hideyo Noguchi” Universidad Autónoma de Yucatán Mérida Mexico; ^4^ Department of Tropical Medicine School of Public Health and Tropical Medicine Tulane University New Orleans LA USA

**Keywords:** adaptive dynamics, domiciliation, generalist parasite, insecticide resistance, local adaptation, microevolution, multihost virulence evolution, Trypanosomatidae

## Abstract

The aetiological agent of Chagas disease, *Trypanosoma cruzi*, is a key human pathogen afflicting most populations of Latin America. This vectorborne parasite is transmitted by haematophageous triatomines, whose control by large‐scale insecticide spraying has been the main strategy to limit the impact of the disease for over 25 years. While those international initiatives have been successful in highly endemic areas, this systematic approach is now challenged by the emergence of insecticide resistance and by its low efficacy in controlling species that are only partially adapted to human habitat. In this contribution, we review evidences that Chagas disease control shall now be entering a second stage that will rely on a better understanding of triatomines adaptive potential, which requires promoting microevolutionary studies and –omic approaches. Concomitantly, we show that our knowledge of the determinants of the evolution of *T. cruzi* high diversity and low virulence remains too limiting to design evolution‐proof strategies, while such attributes may be part of the future of Chagas disease control after the 2020 WHO's target of regional elimination of intradomiciliary transmission has been reached. We should then aim at developing a theory of *T. cruzi* virulence evolution that we anticipate to provide an interesting enrichment of the general theory according to the specificities of transmission of this very generalist stercorarian trypanosome. We stress that many ecological data required to better understand selective pressures acting on vector and parasite populations are already available as they have been meticulously accumulated in the last century of field research. Although more specific information will surely be needed, an effective research strategy would be to integrate data into the conceptual and theoretical framework of evolutionary ecology and life‐history evolution that provide the quantitative backgrounds necessary to understand and possibly anticipate adaptive responses to public health interventions.

## 1 INTRODUCTION

**Figure 1:**
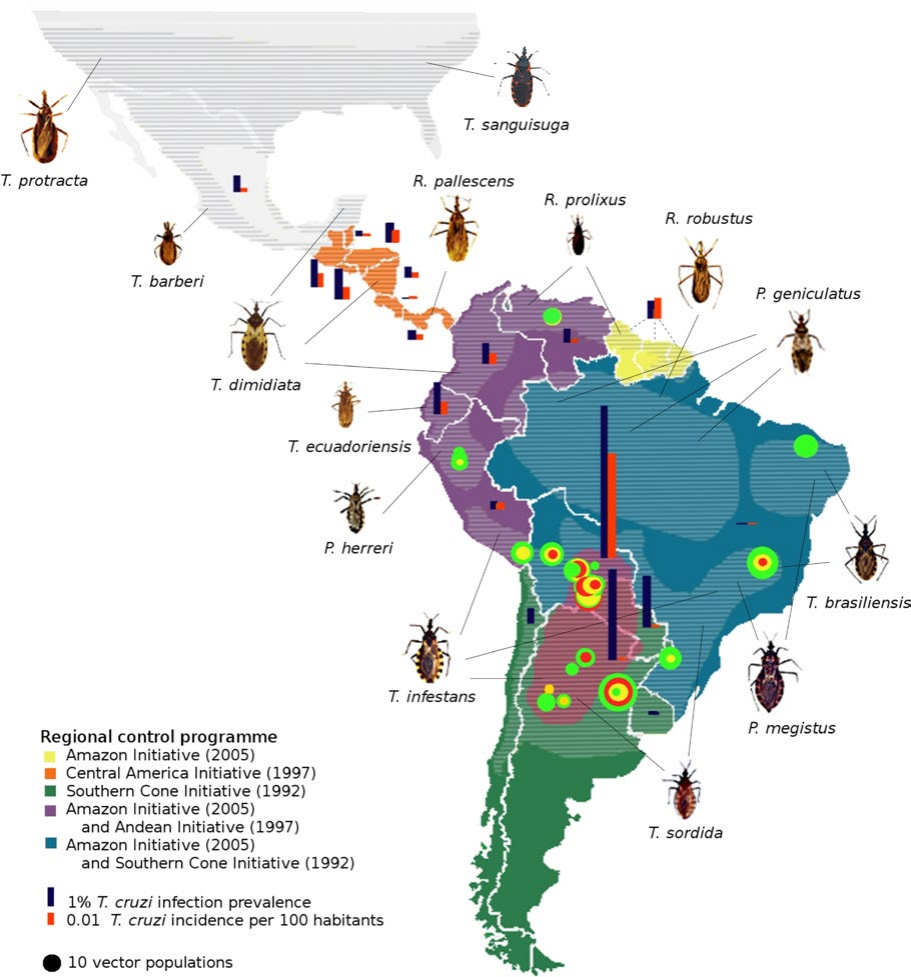
Eco‐epidemiology of Chagas disease. The spatial distributions of the main triatomine vector species appear as shaded areas, while countries endemic for Chagas disease are coloured according to their contribution(s) to the main regional control programs initiated in the 1990–2000s. The last WHO's statistics about national prevalence and incidence of the disease are given for each of these countries. The nationwide data summarized on this map are also provided in Appendix A in Supporting Information. Green, yellow and red circles stand for susceptible, resistant and highly resistant triatomine populations. The sizes of the circles are proportional to the number of populations of each province where resistance to one or more insecticides has been detected. The highly endemic Gran Chaco eco‐region, where most resistance has been observed so far, is highlighted in red

American trypanosomiasis, also named Chagas disease after the Brazilian physician who first described the trypanosome, its vectors and hosts (Chagas, 1909), is a key human vectorborne zoonotic disease that is endemic in 21 Latin American countries (Figure 1) and the southern United States (Bern, Kjos, Yabsley, & Montgomery, 2011; World Health Organisation, 2014), and it is now also spreading through international migrations into Europe (Perez‐Molina et al., 2011), Canada (Schipper, McClarty, McRuer, Nash, & Penney, 1980), New Zealand and Australia (Jackson, Pinto, & Pett, 2014). The trypanosome develops slowly in humans with a brief acute phase followed by long‐lasting chronic conditions characterized by cardiac and/or digestive pathologies leading to variable debilitating and life‐threatening effects (Rassi, Rezende, Luquetti, & Rassi, 2010). The chronicity of the pathogenesis has contributed to make this trypanosomiasis a “silent” disease, thereby delaying major public health initiatives to the 1990s, and it is now associated with a high hidden cost contributing to a global health and economic burden that makes it a major human disease with societal costs similar to those of uterine, cervical and oral cancers (Lee, Bacon, Bottazzi, & Hotez, 2013).

While Chagas disease is becoming part of public health policies in North America (CDC, 2013; PHAC, 2015), Europe (Basile et al., 2011) and Australia (Jackson et al., 2014), the vast majority of the 6–7 million people diagnosed with the disease live in Latin America with a prevalence of infection reaching up to 6.1% in Bolivia and 3.6% in Argentina (World Health Organisation, 2015b), while another 65 million people from the Americas are at risk of infection as they are daily exposed to vector transmission (World Health Organisation, 2014). The disease ecology in these endemic areas is arguably one of the most complex of all human vectorborne diseases whose transmission is further impacted by ongoing socio‐economical changes (Briceño‐León, 2007; Briceño‐León & Méndez Galván, 2007). The causal agent of Chagas disease, the protozoan *Trypanosoma cruzi*, is a genetically diverse parasite able to infect a broad range of vertebrates including over 100 mammal species (Jansen & Roque, 2010; World Health Organisation, 2002). The transmission of *T. cruzi* within its host (meta‐)community is vectored by a large species diversity of bugs of the triatominae subfamily that are generalist haematophagous able to feed on an even broader range of vertebrate species (Gourbière, Dorn, Tripet, & Dumonteil, 2012). The complexity of these eco‐epidemiological networks is such that *T. cruzi* eradication cannot be targeted and that the WHO Generic Roadmap for Neglected Tropical Diseases focuses on the regional elimination of intradomiciliary transmission by 2020 (World Health Organisation, 2015a). In the absence of vaccine and with the poor availability and side effects of available drugs (Chatelain, 2017), the cornerstone strategy to achieve such a goal is triatomine vector control.

This strategy lies at the heart of the WHO policy (World Health Organisation, 1998, 2010a) and of the multinational initiatives that have been launched since the 1990s to reduce human–vector contact at the regional scale, by massive campaigns of indoor insecticide spraying planned across the Andes (Salvatella, 2007), the southern cone (Silveira et al., 2002), Central America (Mancero & Ponce, 2011) and the Amazon region (World Health Organisation, 2005; Figure 1). These efforts have been successful in strongly reducing transmission due to two key “domestic” vectors, that is, *Triatoma infestans* and *Rhodnius prolixus,* that show strong levels of adaptation to human habitat (Waleckx, Gourbière, & Dumonteil, 2015). The interruption of intradomiciliary transmission due to *T. infestans* has been officially certified in various areas/countries, such as in Uruguay (World Health Organisation, 2012), Chile (World Health Organisation, 2000), several states of Brazil (World Health Organisation, 2006), and more recently in Argentinean provinces, and two departments of Southern Peru (World Health Organisation, 2010b). Today, the persistence of vector transmission in many other places (Patterson & Guhl, 2010) raises new issues for the future of Chagas disease's control. These emerging challenges are associated with typical evolutionary processes that could significantly change the local and/or global patterns of the disease epidemiology: (i) the rise of insecticide resistance (Mougabure‐Cueto & Picollo, 2015; Pessoa, Vinãs, Rosa, & Diotaiuti, 2015), (ii) the adaptation of nondomiciliated vectors to the human habitat (Almeida et al., 2009; Reyes‐Lugo & Rodriguez‐Acosta, 2000; Waleckx, Gourbière, et al., 2015), and (iii) the potential evolution of *T. cruzi* virulence (Bull & Lauring, 2014; Pelosse et al., 2013).

In this contribution, we consider each of these three challenges in turn. For each of them, we aim at reviewing the efforts to provide evidences of the underlying evolutionary processes, and to place the control of the disease into an eco‐evolutionary perspective. Along with a synthesis of the existing data, we intend to identify research lines that would significantly enhance our understanding of the evolutionary forces that shape the current and future patterns of *T. cruzi* transmission. We stress that such an eco‐evolutionary knowledge is likely to become increasingly important to sustain the level of control of “domestic” vectors and to tackle the transmission due to nondomiciliated triatomine species, whose epidemiological importance has definitely been uncovered in the last few years (Waleckx, Camara‐Mejia, et al., 2015; Waleckx, Gourbière, et al., 2015).

## 2 TRIATOMINE LIFE‐HISTORY EVOLUTION AND VECTOR CONTROL CHALLENGES

The last 25 years of triatomine control have been designed under the assumption that the evolutionary potential of triatomines would be weak enough for them not to adapt to the new ecological conditions set by control interventions. This adaptive potential was a priori thought to be weak (Gorla, 1994; Schofield & Dias, 1999) for three principal reasons: the low level of triatomine genetic diversity (Mougabure‐Cueto & Picollo, 2015), their long life expectancy (Guhl & Schofield, 1996; Monteiro, Escalante, & Beard, 2001) and the design of the control interventions based on low‐frequency intervention with very high coverage (Bustamante Gomez, Diotaiuti, & Gorla, 2016). We review below field and laboratory studies on the development of insecticide resistance and the adaptation potential of vectors to human habitat as evidences that triatomine adaptive evolution can be much faster than anticipated and may jeopardize control efforts.

### 2.1 Insecticide resistance

The development of insecticide resistance is a major issue wherever chemical control has been intended (Brown, 1986; Liu, 2015; Mallet, 1989; Rivero, Vézilier, Weill, Read, & Gandon, 2010). Populations of key vectors such *Anopheles* and *Culex* mosquitoes have been resistant to insecticides for over 60 years (Gjullin & Peters, 1952; Jones et al., 2012; Ranson & Lissenden, 2016), and similar resistance has also appeared in *Aedes* mosquitoes (Georghiou, 1986; Vontas et al., 2012). Unsurprisingly, given these past experiences, evidences of pyrethroid resistance are mounting in triatomines, especially in *T. infestans* and *R. prolixus* that have been heavily targeted by international control initiatives.

#### 2.1.1 Insecticide resistance in triatomines; where, when, who and to what extent?

**Figure 2:**
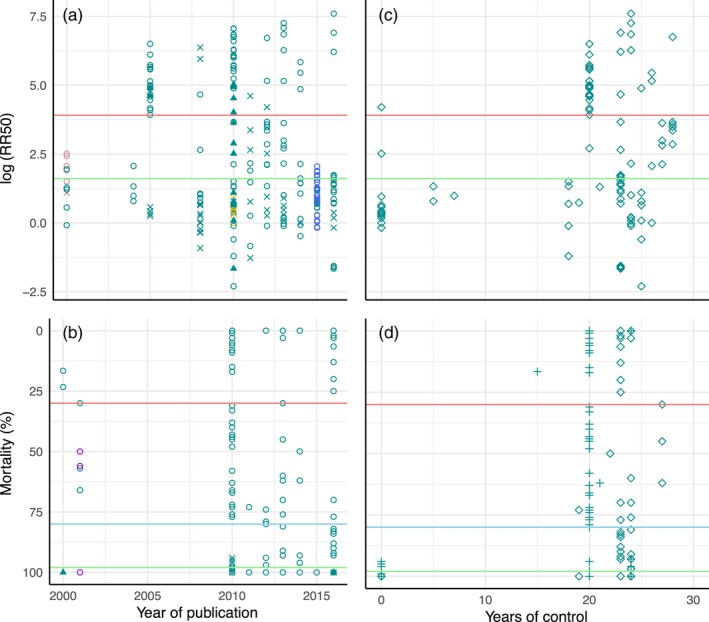
Levels of insecticide resistance reported in triatomines population with respect to their date of detection (a,b) and the number of years of control preceding detection (c,d). The level of resistance is measured as the logarithm of RR_50_ (a and c) or as a percentage of mortality (b and d). The green and pink lines are standard thresholds differentiating between susceptible, resistant and highly resistant triatomines populations. In b and d, no specific status is given to populations lying between the blue and pink lines. Colours indicate different species: green (*Triatoma infestans*), yellow (*Triatoma brasiliensis*), blue (*Triatoma sordida*), pink (*Rhodnius prolixus*) and violet (*Panstrongylus herreri*). Shapes correspond to different types of resistance in a and b; circles (pyrethroids), X (nonpyrethroids) and triangles (insecticide synergy), and they indicate countries in c and d; crosses (Bolivia) and lozenge (Argentina). Data are provided in Appendix B in Supporting Information

Although insecticide resistance had been reported before the major international initiatives were launched (González Valdivieso, Sanchez Diaz, & Nocerino, 1971), its regular assessment began in the mid‐1990s. We compiled measures of pyrethroid resistance in 378 populations studied since then (see Appendix B in Supporting Information), among which 149 (39%) were considered as “resistant.” The most documented foci of resistance are populations of *T. infestans* from northern Argentina (43.6%) and Bolivia (45.6%) with only a few additional cases reported from populations of *T. infestans* in Brazil and Peru, *R. prolixus* in Venezuela, *Triatoma sordida* in Brazil and *Panstrongylus herreri* in Peru (Figure 1). These highly endemic areas being under more investigations than many other places in Latin America, insecticide resistance may actually be spread over a larger spatial range than suggested by Figure 1 (although at different levels, see below). The highest levels of resistance are mostly observed in and around the Chaco region where 79 field populations of *T. infestans* have been shown to be highly resistant (i.e., RR_50_ > 50 or mortality < 30%). In these areas, the level of resistance can be up to 2,000 times higher than the level in reference strains (Sierra, Capriotti, Fronza, Mougabure‐Cueto, & Ons, 2016), and it has already led to control failures (Picollo et al., 2005). Interestingly, we could not detect any temporal change in the levels of insecticide resistance reported since 2000 (Figure 2a,b) or with the number of years of control (Figure 2c,d). This suggests, as previously mentioned (Bustamante Gomez et al., 2016), that natural tolerance was already present to various levels at a large scale, although the highest levels remain concentrated in and around the Chaco area, where resistant and susceptible individuals still coexist at the local scale of a village or even a single household (Germano, Picollo, & Mougabure‐Cueto, 2013). The majority of resistant populations of *T. infestans* were found resistant to deltamethrin (73.9%) with high levels of resistance also observed to other pyrethroids in Argentinian and Bolivian populations (Figure 2c,d), but there currently is little evidence of multiple resistances (Fabro et al., 2012; Germano & Picollo, 2014; Yon et al., 2004).

#### 2.1.2 A priori evolutionary thoughts and evolutionary questions

Insecticide resistance has emerged in triatomines, according to the general trend observed in many insects (Mallet, 1989; Rivero et al., 2010) and despite the a priori evolutionary thoughts mentioned above. Whether the massive spraying of pyrethroids through international initiatives was worth is out of question given the success in controlling some of the most epidemiologically relevant triatomine populations. However, the evidences above raise issues about the sustainability of such vector control. We now need to reconsider our initial evolutionary thoughts in the light of more quantitative data and concepts to characterize triatomines’ true evolutionary potential and possibly identify evolution‐proof spraying strategies that will minimize the development of insecticide resistance while still providing efficient vector control.

##### Genetic diversity and evolutionary potential

That triatomines have a low adaptive potential due to their low genetic diversity is a statement that should be considered with caution. The correlation between genetic diversity measured at neutral molecular markers (typically used in triatomine genetics, see Gourbière et al., 2012 for a review) and short‐term adaptive potential is indeed highly debatable, mostly because such markers retain information from a tiny part of the genome and as they can lose genetic variability at a very different rate than adaptive loci (Reed & Frankham, 2001). By contrast, the ability to adapt to rapidly happening environmental changes induced by human activities is increasingly recognized as a complex combination of genetic and epigenetic factors (Fernández et al., 2014; Rey, Danchin, Mirouze, Loot, & Blanchet, 2016), and it has been hypothesized that epigenetic could contribute to triatomine speciation (Costa et al., 2016; Dujardin, Costa, Bustamante, Jaramillo, & Catala, 2009). Interestingly, it has recently been shown that the spatial distribution of highly resistant populations coincides with the distribution of an intermediate cytogenetic group of *T. infestans* and further correlates with local environmental variables (Bustamante Gomez et al., 2016). This environmental determinism could, as suggested by the authors, be the outcome of an evolutionary dynamics involving some trade‐offs between resistance genes and key ecological traits. Alternatively, it could reflect the effects of abiotic factors on the epigenetic components and/or the role of transposable elements in the regulation of gene expression (Rey et al., 2016). The importance of the regulation of (detoxification) gene expression was indeed recently proposed to explain that populations with similar frequencies of kdr mutations affecting the target sites of pyrethroid present different levels of resistance (Sierra et al., 2016). Meanwhile, it has been suggested that today's patterns of pyrethroid resistance reflect the existence of “naturally tolerant populations” of *T. infestans* rather than selective sweeps associated with insecticide spraying (Mougabure‐Cueto & Picollo, 2015). This is indeed consistent with the reports of resistance profiles in sylvatic populations (Bustamante Gomez et al., 2016; Depickère et al., 2012; Roca‐Acevedo et al., 2011), the broad spatial distribution of resistance (Figure 1, Mougabure‐Cueto & Picollo, 2015) and the apparent absence of temporal changes in the observed levels of resistance (Figure 1b). Undoubtedly, triatomine insecticide resistance is a complex and plastic trait as it involves different mechanisms (González Audino, Vassena, Barrios, Zerba, & Picollo, 2004; Mougabure‐Cueto & Picollo, 2015; Roca‐Acevedo, Picollo, Capriotti, Sierra, & Santo‐Orihuela, 2015) with a polygenic determinism (Pessoa et al., 2015). This trait shall now be investigated through functional genomics approaches (as genuinely initiated by Traverso et al., 2017), if one is to uncover the true nature of its variations, and propose some mechanistic explanations to the intriguing associations of the highest level of resistance with a specific genetic background and environmental variables (Bustamante Gomez et al., 2016).

##### Fitnesses are composite measures

A critical difficulty in assessing our evolutionary a priori and the evolutionary questions raised above is the lack of integrative measures that would allow comparing the fitness of resistant and susceptible individuals with respect to trait values over their entire life cycle, that is, the duration, survival and fecundity rates of each developmental stages, and the effect of insecticide on those different traits. Most experimental studies indeed focus on a single stage and typically measure effects of insecticide on survival of eggs (6%), first and fifth instars (86%, 4%) or adults (4%) of triatomines caught in their domestic (35%), peridomestic (58%) and sylvatic (7%) habitats (data from Appendix B in Supporting Information). Some pioneering attempts provide interesting estimates of both fecundity and longevity of the different stages from colonies with different levels of resistance to deltamethrin (Germano & Picollo, 2014; Pires, Barbosa, & Diotaiuti, 2000). More resistant populations laid a lower number of eggs (Germano & Picollo, 2014) of higher weight (Pires et al., 2000). Meanwhile, those studies show an extension of egg or duration of instar stages in resistant individuals with compensation in others, so that there is overall no effect of resistance on the length of the complete life cycle. Although these lower reproductive outputs and developmental delays were interpreted as costs of resistance, they only affect the fitness of resistant and susceptible individuals when exposed to insecticide. Just as Germano and Picollo's (2014) data provide evidences of trade‐off between the duration of different instar stages, insecticide resistance could be linked through trade‐offs with the rates of survival and adult life stage, but this was not evaluated. Expressions of fitness with respect to life‐history traits of individuals with developmental stages have been developed for a long time (Caswell, 2001, chapters 3–4) allowing to account for various trade‐offs between such traits (Roff, 2002, chapter 4; Roff, 2010, chapters 2–3). To provide quantitative studies about the evolution of resistance in triatomines now requires such integrative fitness measures to be calculated for resistant and susceptible individuals in both treated and nontreated conditions as the evolution of resistance usually involves some form of spatially heterogeneous or temporally variable selection with a selective disadvantage of resistant individuals in nontreated environment. Triatomines are typically exposed to lethal doses inside domiciles and sublethal doses in peridomiciles (Bustamante Gomez, Caldas Pessoa, Luiz Rosa, Espinoza Echeverria, & Gonçalves Diotaiuti, 2015; Fronza, Toloza, Picollo, Spillmann, & Mougabure‐Cueto, 2016). According to existing models, sublethal doses are likely to limit the evolution of resistance as selective pressures are then relaxed on earlier life stages and mostly affect the older and more infected vector individuals (Read, Lynch, & Thomas, 2009). Accordingly, the peridomestic habitat could provide a reservoir for re‐infestation (Cecere et al., 2013; Gürtler, 2009) but with less resistant individuals, which could potentially impede the spread of resistant genes. Similarly, the effect of temporal heterogeneity in control interventions could be assessed. In such context, an interesting point raised by Germano and Picollo (2014) is that insecticide spraying might be seen as a strong random environmental variation. Such variability is well known to select for bet‐hedging strategies and adaptive developmental delays (Gourbière & Menu, 2009 and references therein) that could explain prolonged developmental times in triatomine nymphs (Menu, Ginoux, Rajon, Lazzari, & Rabinovich, 2010).

### 2.2 Domiciliation

There is a large diversity of triatomine species (Gourbière et al., 2012; Lent & Wgodzinsky, 1979; Schofield & Galvão, 2009) whose epidemiological importance is linked to their level of adaptation to human environment, that is, their level of domiciliation. Understanding the ability of triatomine species/populations to occupy wild and/or human habitat and its evolvability is thus essential in both optimizing today's control strategies and anticipating future epidemiological trends.

#### 2.2.1 Domiciliation, transmission and vector control

The epidemiological importance of triatomine species/populations is linked to their level of domiciliation as the latter defines the level of human/vector contacts (Dujardin, Schofield, & Panzera, 2002), which in turn has important implications for the design and efficacy of vector control interventions (Abad‐Franch, 2016; Waleckx, Gourbière, et al., 2015) that are summarized in Table 1. Insecticide spraying in human dwellings is likely to be successful only in settings where the vector species targeted lives exclusively in human structures and there is no sylvatic population, which can act as a source of re‐infestation (case 1 in Table 1). This is the case where vector species have been introduced in the domestic environment out of their place of origin, such as *T. infestans* in Brazil or *R. prolixus* in Central America, which have been successfully eliminated after chemical control (Hashimoto & Schofield, 2012; Schofield, Jannin, & Salvatella, 2006; Silveira & Vinhaes, 1999). In alternative settings, where the vector species is native and maintains sylvatic populations, its elimination is actually impossible, and insecticide spraying has been shown to be much less effective. For example, while being the species best adapted to human habitat and despite years of vector control by insecticide, *T. infestans* persists in human dwellings in Bolivia, where various sylvatic populations have been reported (case 2 in Table 1, Torrico, 1946; Bermudez, Balderrama, & Torrico, 1993; Noireau et al., 1997; Buitrago et al., 2010; Waleckx et al., 2011, 2012). Part of the observed re‐infestation of houses can be attributed to the dispersal of bugs from sylvatic populations (Brenière et al., 2013), and a combination of chemical control with alternative strategies to impede the entry of sylvatic vectors should be preferred to chemical control alone in such areas. Another example comes from the Yucatan peninsula (Mexico), where *Triatoma dimidiata* is sylvatic and merely enters human dwellings but has not been able to adapt yet to establish colonies in this habitat (Case 3 in Table 1, Dumonteil et al., 2002; Gourbière, Dumonteil, Rabinovich, Minkoue, & Menu, 2008; Dumonteil, Ramirez‐Sierra, Ferral, Euan‐Garcia, & Chavez‐Nunez, 2009; Waleckx, Pasos‐Alquicira, Ramirez‐Sierra, & Dumonteil, 2016). In this setting, chemical control is totally ruled out (Barbu, Dumonteil, & Gourbière, 2009), and alternative strategies, such as the installation of insect screens, are more likely to be effective and sustainable (Barbu, Dumonteil, & Gourbière, 2011; Barbu et al., 2009; Waleckx, Camara‐Mejia, et al., 2015; Table 1). Triatomine species considered in the process of domiciliation/domestication, such as *Triatoma sherlocki* (Almeida et al., 2009) or *Panstrongylus geniculatus* (Reyes‐Lugo & Rodriguez‐Acosta, 2000; Waleckx, Gourbière, et al., 2015), illustrate entomological situations similar to *T. dimidata* in Yucatán. Finally, in places where primary vector species can be successfully removed, the control of *T. cruzi* transmission can also be challenged if secondary vectors exist in ecotopes surrounding treated dwellings (case 4 in Table 1). For example, in Brazil, the interruption of chemical treatment after successful elimination of *T. infestans* has been followed by recolonization by native species such as *Triatoma brasiliensis* in the north‐eastern region or *Panstrongylus megistus* in the coastal areas (Silveira & Vinhaes, 1999). A combination of insecticide spraying and strategies targeting dispersal may thus also be necessary in such cases.

**Table 1:** Control interventions and potential evolutionary issues according to the adaption of vector populations to their typical ecotopes

Case	Vector populations		Control interventions					Potential evolutionary issues			
	Domicile and peridomicile	Sylvatic ecotopes	Goals	Chemical control	Physical barrier	House improvement	Entomological monitoring	Insecticide resistance	Behavioural changes	Domiciliation	Section
1	Yes	No	Elimination	X			X	X	X		2.1
2	Yes	Yes	Elimination in human habitat Limit dispersal into domiciles	X	X	X	X	X	X		2.1, 2.2
3	No	Yes	Limit dispersal into domiciles		X	X			X	X	2.2
4	Yes	Yes—secondary vectors	Elimination in human habitatLimit dispersal into domicilesLimit adaptation of secondary vectors	X	X	X	X	X	X	X	2.1, 2.2

#### 2.2.2 Triatomine domiciliation potential

Although it is generally thought that anthropogenic pressure and damage to triatomine biotopes promote dispersion towards human dwellings and domiciliation of sylvatic triatomines, the evolutionary processes and driving mechanisms of adaptation to human environments remain poorly understood. On the one hand, taking into account the high morphologic plasticity of Triatominae associated with a rapid adaptation to different ecotopes (Dujardin, Steinden, Chavez, Machane, & Schofield, 1999; Dujardin et al., 2009), as well as the high diversity of human habitats, and the catholic feeding habits observed for many species of triatomines (Rabinovich et al., 2011), it is safe assuming that any triatomine population can infest human dwellings in particular settings. On the other hand, only a small fraction of triatomines are able to establish sustainable domestic colonies there, suggesting that these have evolved a set of traits which confers the ability to exploit domestic habitat (Abad‐Franch & Monteiro, 2007). According to Schofield, Diotaiuti and Dujardin (1999), domiciliation involves both genetic and phenetic simplification as a result of a strong inside dwelling intraspecific competition after invading populations reach the carrying capacity of the domestic habitat. They speculated that such simplified genotypes would be the most efficient in obtaining blood and avoiding to “waste energetic resources to produce genes or gene products that may not be used.” While such adaptive view has been influential, including for vector control (see 2.1), its ecological foundations remain loosely documented. First, there is no quantitative evidence of the specificity and presumably higher stability of the “domestic” habitat. If there are probably less demographic changes of the blood hosts than in sylvatic ecotopes, and maybe less temperature variations (this clearly depends on human dwelling type and wild nests we are comparing), humans also tend to remove insects from its habitat, so stability may be very subjective. Second, the link between changes in ecological traits and selective advantage to triatomines in a domestic habitat is lacking. The authors used an eclectic mix of evidences to back their proposal about morphological and genetic changes observed in the transition from natural to artificial habitat. These included a progressive simplification of the sensory system in accordance with increasing habitat stability, a relaxed bilateral symmetry, a general reduction in body size, mainly in female bugs, leading to a corresponding reduction in sexual dimorphism (as females are on average larger than males in Triatominae), a decrease in total DNA per cell, a reduction in variability and polymorphism of isoenzymes, and a reduction in gene sequence variability (Schofield et al., 1999). Unfortunately, these observations have not been accompanied with any studies on the fitness of these different morpho‐ and genotypes in artificial environments to effectively link simplified genotypes or phenotypes with increased competitive ability in domestic habitats. In addition, most of the observed changes have not been supported by subsequent studies. For example, while Panzera et al. (2004) reported a reduction in DNA content in *T. infestans* from the Andes to the lowlands in South America, Hernandez, Abrahan, Moreno, Gorla, and Catala (2008) reported an increasing complexity of the antennal sensilla pattern of the same insects from the lowlands to the Andes. Antennal sensilla patterns have also been shown to depend on many parameters including species, populations of a same species, sex and microhabitat (Arroyo, Esteban, Catala, & Angulo, 2007; Catala, Maida, Caro‐Riano, Jaramillo, & Moreno, 2004; Dujardin et al., 2009; Hernandez et al., 2008), so that a simplification of the sensory system in the transition from sylvatic to domestic habitat (which provide plenty of different microhabitats) is a too simplistic idea and cannot be established as a rule. Similarly, the reduction in sexual dimorphism, which has been proposed as a marker of domiciliation in Triatominae (Dujardin et al., 1999), or the levels of fluctuating asymmetry—which is expected to be lower in stable habitats (Marquez & Saldamando‐Benjumea, 2013; Nattero, Dujardin, del Pilar Fernández, & Gürtler, 2015) while the model of domiciliation of Triatominae mentioned above paradoxically predicts a relaxed bilateral symmetry as a consequence of habitat stability leading to a demographic increase and subsequent competition for food (Schofield et al., 1999), do not show consistent enough patterns, and should also be interpreted with caution (Dujardin et al., 1999; Marquez & Saldamando‐Benjumea, 2013; Nattero et al., 2015; Sandoval Ramirez et al., 2015). Consequently, the morphological characters generally studied in Triatominae appear too variable to trustworthy be used to infer the level of domiciliation of triatomine species/populations or as markers of potential for domiciliation.

As mentioned above, a high plasticity is also observed in terms of blood hosts for many triatomine species (Rabinovich et al., 2011). This feature suggests that triatomines can easily adapt to new blood sources if their natural hosts disappear (as can occur when human and its domestic animals invade new areas), and potentially use human/domestic animals without a real evolutionary cost. Some studies have measured different life‐history traits of triatomines in relation to different blood hosts (Emmanuelle‐Machado et al., 2002; Gomes, Azambuja, & Garcia, 1990; Guarneri, Araujo, Diotaiuti, Gontijo, & Pereira, 2011; Guarneri, Pereira, & Diotaiuti, 2000; Lunardi, Gomes, Peres Camara, & Arrais‐Silva, 2015; Martinez‐Ibarra, Grant‐Guillen, Nogueda‐Torres, & Trujillo‐Contreras, 2004; Martinez‐Ibarra et al., 2006; Medone, Balsalobre, Rabinovich, Marti, & Menu, 2015; Nattero, Leonhard, Rodriguez, & Crocco, 2011; Nattero, Rodriguez, & Crocco, 2013), as well as blood host preferences (Crocco & Catala, 1997; Gürtler et al., 2009; Jiron & Zeledon, 1982). This kind of studies can shed light on the attractiveness of human and/or domestic animals as blood hosts, as well as the performances and advantages/disadvantages to feed on them, and can thus help predicting the potential for domiciliation of different triatomine populations (Guarneri et al., 2011). Nevertheless, results need again to be interpreted with caution and extrapolation to natural situations may be difficult, as many traits affect fitness and results can depend on the traits studied. For example, while Nattero et al. (2011) suggested a better reproductive success for *T. infestans* feeding on mammalian rather than avian blood, Medone et al. (2015) reported a better population growth for *T. infestans* feeding on hen rather than human blood. Moreover, while better performances of the studied traits when feeding on human/domestic animals may suggest that a triatomine population will gain advantage to invade human dwellings, it will not necessarily happen. Many factors cannot be reproduced in the laboratory, including host behaviour in its natural habitat. Inversely, this is not because lower performances are observed when feeding on human that invasion of human dwellings will not happen. The association with blood hosts depends on the benefit/cost ratio for triatomines, and a nonoptimal association can be preferred or even vital on some occasions, as evidenced by the fact that feeding patterns are modified depending on the availability and density of vectors and hosts (Gürtler et al., 2009). In such a context, data‐driven modelling approaches could be used to concomitantly derivate fitness measures of vectors and help predicting domiciliation potential by expanding standard evolutionary ecology theories rooted in source–sink dynamical models (Gourbière & Gourbière, 2002; Nouvellet, Cucunubá, & Gourbière, 2015; Rascalou, Pontier, Menu, & Gourbière, 2012).

## 3 *TRYPANOSOMA CRUZI* DIVERSITY AND VIRULENCE EVOLUTION

While triatomine vectors blood feed on a variety of vertebrate hosts including amphibians, reptiles, birds and mammals, *T. cruzi* infection is restricted to mammalian species. For example, complement‐mediated lysis of the parasites rapidly occurs in birds, making them refractory to *T. cruzi* infection (Lima & Kierszenbaum, 1984; Minter‐Goedbloed & Croon, 1981). *Trypanosoma cruzi* can nonetheless be considered a very generalist parasite, able to infect a large range of mammalian species covering very different orders, including Marsupialia, Rodentia, Lagomorpha, Chiroptera, Carnivora and Primata. While the infection in these different orders may have variable outcomes, there are also many common features in the acquisition and the within‐host dynamic of *T. cruzi* that may contribute to explain its overall high diversity and low virulence that are described below. We refer here to virulence as a parasite‐induced loss of host's fitness. This broad definition encompasses both the empirical assessments of *T. cruzi* sublethal effects (see 3.1) and the theoretical acceptation of virulence as a rate of pathogen‐induced host mortality (see 3.2).

### 3.1 *T. cruzi* diversity and its determinants

**Figure 3:**
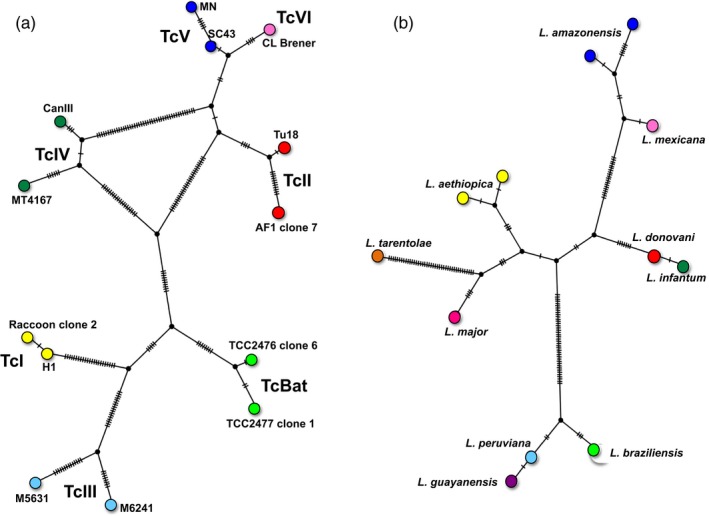
Genetic diversity of *Trypanosoma cruzi* and *Leishmania* spp. TCS haplotype networks for *T. cruzi* DTUs (a) and *Leishmania* species (b) were constructed based on sequence alignments of the mini‐exon intergenic sequence and HSP70 gene, respectively, using PopArt. *T. cruzi* strains and corresponding DTUs (TcI to TcVI and TcBat) are indicated, as well as *Leishmania* species. Ticks on network branches indicate the number of mutations from one haplotype to the next. The mini‐exon sequences were 210–250 bp in length, with 122 segregating sites, and 99 parsimony informative sites for the *T. cruzi* network. *Leishmania* HSP70, sequences were 1,245 bps in length, with 110 segregating sites, and 79 parsimony informative sites

*Trypanosoma cruzi* represents one of the best model organisms following a predominant clonal evolution model, with rare recombination and/or hybridization events (Tibayrenc & Ayala, 2015). The high genetic diversity of the parasite is believed to have arisen from this clonal model, leading to its subdivision into seven discrete typing units (DTUs TcI to TcVI and Tcbat), which are highly stable across the Americas and over time (Zingales et al., 2009, 2012), and correspond to a (near‐) clade genetic structuration of the parasite as a species (Tibayrenc & Ayala, 2015). This level of genetic diversity among *T. cruzi* DTUs is comparable to that observed among some *Leishmania* species (Yeo et al., 2011; and Figure 3). While TcI to TcIV are considered monophyletic and ancient clades, TcV and TcVI are more recent natural hybrids of TcII and TcIII DTUs (Lewis et al., 2011). However, with improved genotyping methods, a growing number of studies suggest that recombination and hybridization may be much more frequent than previously acknowledged, challenging the current model of clonal evolution (Messenger & Miles, 2015), and more studies will be required to clarify this issue.

Many studies have attempted to associate this genetic variability of the parasite with specific vertebrate hosts and transmission cycles, restricted geographic distribution of strains and DTUs, and the biological and clinical characteristics of the infection (Messenger, Miles, & Bern, 2015). For example, it was believed that TcI DTU was largely predominant in Mexico and to some extent in Central America (over 95% of the strains), but recent studies have documented the presence of non‐TcI parasite strains in triatomines from different regions in Mexico and Central America at high frequencies (Ibanez‐Cervantes et al., 2013; Pennington, Paiz, Grajeda, & Cordon‐Rosales, 2009; Torres‐Montero, López‐Monteon, Dumonteil, & Ramos‐Ligonio, 2012), and a high proportion of patients appear to be infected with non‐TcI parasite strains (Risso et al., 2011). Similarly in the southern United States, initial work reported only TcI and TcIV DTUs (Roelling et al., 2008), but recent studies also indicate the presence of TcII DTUs in rodents (Herrera, Licon, Nation, Jameson, & Wesson, 2015), as well as the predominant presence of TcII‐TcV‐TcVI in autochthonous human cases (Garcia et al., 2017). Together, these observations indicate clearly that *T. cruzi* genotype distribution in Central and North America is still poorly understood (Brenière, Waleckx, & Barnabe, 2016).

Other studies have suggested an association between *T. cruzi* DTUs and their biological properties and the pathogenesis of *T. cruzi* infection (Andrade & Magalhães, 1997; Carneiro, Romanha, & Chiari, 1991; Cencig, Coltel, Truyens, & Carlier, 2013; Lewis, Francisco, Taylor, Jayawardhana, & Kelly, 2016), raising hopes that infection outcome in humans may be predicted by the infecting DTU. However, these studies all suffer from the examination of a very limited number of strains, making such generalization poorly supported. Genotyping studies in large cohorts of patients with different clinical profiles may provide stronger evidence of such an association, but so far have been unable to provide evidence of associations between clinical outcomes and parasite DTUs (Martinez‐Perez et al., 2016; Perez‐Molina et al., 2014; Zafra, Mantilla, Jacome, Macedo, & Gonzalez, 2011), and contrasting results associating hosts and parasite DTUs have been described (Cura et al., 2010; Llewellyn et al., 2009; Monje‐Rumi et al., 2015). A major limitation is that current PCR genotyping methods are poorly sensitive for the successful genotyping of parasites in chronically infected patients because of the very low amounts of parasite DNA present, so that genotypes can only be identified in about 50% of the patients (Cura et al., 2012, 2015; Garcia et al., 2017). The used of multiple consecutive blood samples from patients may increase the success rate of the genotyping to up to 70% of the patients (Martinez‐Perez et al., 2016). Importantly, DTU classification is mostly based on the analysis housekeeping gene sequences such as ribosomal RNA genes and metabolic enzymes, that are unlikely to have a strong relevance for the virulence of the parasite, and alternative genetic markers derived from proteins playing a key role in the biological events of *T. cruzi* infection may be more likely to reveal potential associations between genotypes and the biological characteristics of parasite strains (De Pablos & Osuna, 2012).

Recent studies are also focusing on the genetic diversity within DTUs (Brenière et al., 2016). Within hosts of the order Didelphimorpha, haplotype and nucleotide diversity of TcI are of the same magnitude as within all other wild mammals combined, strongly suggesting a longer and closer association between *T. cruzi* and Didelphimorpha (Brenière et al., 2016). These observations suggest a genetic structuration of *T. cruzi* influenced by the coevolution and adaptation of the parasite to specific hosts and transmission cycles, which should be examined further to establish the epidemiologic relevance of specific *T. cruzi* genotypes in human Chagas disease. Nonetheless, ecological host fitting may also represent an important mechanism allowing the parasite to maintaining its genetic diversity and ample range of vertebrate hosts (Agosta, Janz, & Brooks, 2010). Indeed, under this scenario, the phenotypic plasticity of parasites allows them to be pre‐adapted to new resources/hosts. A detailed genetic study revealed a limited gene flow between *T. cruzi* from different transmission cycles and a low level of genetic structure among strains from similar ecotopes in Bolivia, taken as evidence of ecological host fitting underlying the diversification of the parasite (Messenger et al., 2015). It is in fact likely that the complex interactions between *T. cruzi* and its vertebrate hosts are shaped by a combination of multiple nonexclusive evolutionary and ecological mechanisms (Agosta et al., 2010), and untangling their relative contributions remains challenging. Further studies are thus needed to clearly understand the different evolutionary forces underlying *T. cruzi* parasite diversity and the epidemiology of Chagas disease.

Finally, current approaches focus almost exclusively on detecting the dominant parasite genotype in biological samples (triatomine vectors and vertebrate hosts), so that the multiclonality of infections is overlooked. One of the rare studies analysing the multiclonality of *T. cruzi* infection in mammalian hosts revealed concurrent infections with up to 10 parasite genotypes in the same host and suggested the occurrence of diversifying selection on the parasite (Llewellyn et al., 2011). The analysis of sequence variation in GP63, a major surface glycoprotein involved in parasite infectivity to mammalian cells, revealed a large number of gene variants present in Chagasic patients, also suggesting a large parasite genetic diversity within hosts (Llewellyn et al., 2015). However, because this gene is present in multiple copy number in the parasite genome, the clonal structure of the infecting parasite population could not be established in that study. Also, no significant associations could be made between GP63 antigenic diversity and epidemiological and clinical parameters. However, strong selection could be evidenced, with a significant excess of nonsynonymous substitutions, suggesting that this may be a key factor contributing to parasite genetic diversity (Llewellyn et al., 2015). The multiclonality of infection in humans is also evidenced in longitudinal studies of patients follow‐up, a large proportion of which present changes in the dominant DTU identified before and after drug treatment, suggesting that cryptic mixed infections and heterogeneous drug response may be occurring (Martinez‐Perez et al., 2016). Multiclonal infections may thus be the norm rather than the exception, and the interactions among parasite genotypes within hosts are still mostly unknown, although they may lead to different infection profiles (Ragone et al., 2015) and have important evolutionary implications. Overall, a better understanding of the multiclonality of infection is clearly needed for a detailed understanding of parasite infection dynamics and of the evolutionary forces that modulate parasite genetic diversity (Lewis & Kelly, 2016).

### 3.2 The evolution of *T. cruzi* virulence

#### 3.2.1 The secret and lazy life‐history of *T. cruzi*; silent, dormant and persistent

The transmission and the epidemiology of *T. cruzi* are influenced by the long‐term dynamics of host infection that is associated with the very peculiar life history of this protozoan parasite. Although oral transmission has been reported in humans and nonhuman hosts (Henriques, Henriques‐Pons, Meuser‐Batista, Ribeiro, & de Souza, 2014; de Noya & González, 2015; Sánchez & Ramírez, 2013; Silva‐dos‐Santos et al., 2017), typical infection starts with an acute phase where the parasite enters the host through microlesions of the skin before multiplying in the bloodstream and spreading to other tissues. In humans, this infective stage lasts for 4–8 weeks and it is usually asymptomatic, although it might present as a febrile illness with local reaction at the site of infection (Rassi et al., 2010). Most individuals survive this acute stage and enter a chronic asymptomatic stage. The blood parasitemia then falls, and *T. cruzi* parasitizes myocytes located within cardiac, skeletal or smooth muscle tissues. Parasite persistence may then involve different mechanisms that potentially include continuous intracellular replication, dormant forms of *T. cruzi* and intermittent reactivation (Lewis & Kelly, 2016). Over the course of infection, those combine to induce local and global inflammatory responses that, together with the parasite‐mediated autoimmune response (Teixeira, Nascimento, & Sturm, 2006), determine the timing and intensity of Chagas disease pathology. Most patients actually never develop any clinical symptoms, and only 30%–40% of infected humans show cardiac (20%–30%), digestive (10%–15%) or cardiodigestive pathological manifestations that typically emerge 10–30 years after initial infection (Rassi et al., 2010). Overall, *T. cruzi* shall thus be considered of low virulence as in most infections, it persists inside its host over its lifespan causing little harm. Although our knowledge on the pathogenesis of *T. cruzi* in nonhuman hosts is limited, the dynamics of experimental infections in mouse (Henriques et al., 2014; Vorraro et al., 2014) and the high prevalence of infection in various reservoirs (Cortez et al., 2006; Herrera et al., 2005; Orozco et al., 2013) suggest that *T. cruzi* infection may there rely on the same pillars: silence, dormancy and persistence. In an evolutionary context, one spontaneously wonders why no strain of *T. cruzi* was able to evolve a higher level of virulence? Basic ingredients of existing theories suggest that it should indeed have done so. Vectorborne pathogens are expected to exhibit stronger virulence than directly transmitted diseases as their impact on host mobility is not associated with a reduction in transmission that remained possible through the vectors (Boots & Sasaki, 1999; Ewald, 1993, 2004; Gandon, 2004). Although very high levels of vector domiciliation can be associated with severe restriction of gene flow and dispersal (even at the scale of individual households, Noireau, Zegarra, Ordonez, Gutierrez, & Dujardin, 1999; Brenière, Lopez, Vargas, & Barnabé, 1997; Brenière et al., 1998), in most places triatomines disperse between human dwellings and surrounding habitats so that the parasite is likely to be spread out of infected hosts to reach various other host individuals and/or species, which should have provided many opportunities for virulence to evolve to a higher level. In addition, *T. cruzi* shows a high level of genetic diversity with several well‐identified phylogenetic lineages and an important heterogeneity inside of each lineage (see above), which has been shown to favour the evolution of virulent strains because of within‐host competition (see Alizon, Hurford, Mideo, and Van Baalen (2009) for a review). The restrained life history of *T. cruzi* is puzzling, and one can only hope to resolve this apparent paradox with a better understanding of the evolutionary pressures that shape its evolution.

#### 3.2.2 Evolutionary paradox or evolutionary adaptation?

There is virtually no effort specifically devoted at identifying the key factors driving the evolution of *T. cruzi* virulence through classical theoretical approaches. The only contribution sets under the light of life‐history evolution is by Pelosse et al. (2013), and it shows that vector's risk spreading strategies can speed up the invasion of virulent strains in a stochastically variable environment. Although truly inspiring, this paper relies on numerical analyses that do not seek to provide the simple (analytical) foundations for a broad theory of *T. cruzi* virulence evolution. We thus focus here on exploiting theoretical results that were obtained in the context of *T. cruzi* eco‐epidemiology with the aim of illustrating that they can readily be used to provide general predictions on *T. cruzi* adaptive evolution, which will fit into the overall theory of virulence evolution. Expressions of the basic reproductive number (*R*_0_) have been developed to look at *T. cruzi* population dynamics and can indeed be used as fitness measures in a simple “optimality” approach (Bull & Lauring, 2014; Parker & Smith, 1990). From the expressions of *R*_0_ found in the literature (see Appendix C in Supporting Information), we established a “core” *R*_0_:

$$R_{0}=\sqrt{\frac{\beta_{h}\cdot c_{h}\left(N_{h},N_{v}\right)}{\left(\mu_{h}+\alpha_{h}\right)}\cdot \frac{\beta_{v}\cdot c_{v}\left(N_{h},N_{v}\right)}{\left(\mu_{v}+\alpha_{v}\right)}},$$

that gives the rate of emergence of a *T. cruzi* strain according to the (i) per contact probabilities of transmission from vector/host to host/vector (β_*h*_, β_*v*_), (ii) contact rates between susceptible host/vector and infected vector/host (*c*_*h*_(*N*_*h*_, *N*_*v*_), *c*_*v*_(*N*_*h*_, *N*_*v*_)), death rates of host and vector (μ_*h*_, μ_*v*_), and (iii) virulence, that is, additional mortality to host and vector (α_*h*_, α_*v*_). Assuming a typical transmission–virulence trade‐off, it comes β_*h*_ = ε·α_*h*_/(1 + α_*h*_), ε stands for the maximal probability of stercorarian transmission, and a linear relationship between virulences, that is, α_*v*_ = ν·α_*h*_. An analytical expression of the optimal level of virulence to host ($\alpha_{h}^{*}$) can then be derived searching for the maximal value of *R*_0_:

$$\alpha_{h}^{*}=\sqrt{\frac{\mu_{h}\mu_{\nu }}{\mu_{\nu }+\nu (1+\mu_{h})}},$$

that allows establishing the first basic predictions about the effects of key features of *T. cruzi* transmission on the evolution of its virulence.

A first specificity of *T. cruzi* life history comes from its stercorarian mode of transmission. The pathogen multiplies within the gut of triatomines, but it is not able to reach their salivary glands so that transmission occurs through the vector faeces. The per bite probability of transmission from infected vector to susceptible host is thus unusually low, with estimates of the orders of 10^−4^ to 10^−3^ on humans (Nouvellet, Dumonteil, & Gourbière, 2013; Rabinovich, Wisnivesky‐Colli, Solarz, & Gürtler, 1990) and 10^−4^ to 10^−2^ on reservoirs (Basombrío et al., 1996; Rabinovich, Schweigmann, Yohai, & Wisnivesky‐Colli, 2001). According to the expression of $\alpha_{h}^{*}$, this low probability of transmission is expected not to have any impact on the evolution of *T. cruzi*, basically because the (in)efficacy of the transmission processes (ε) is similar for all strains. Such (in)efficacy has been related with the time elapsing between feeding and defecation, and a “defecation index” allows comparing the transmission potential of different triatomine species (Loza‐Murguía & Noireau, 2010; Reisenman, Gregory, Guerenstein, & Hildebrand, 2011). Hypothetically, different strains could thus be transmitted with different efficacies if they had different capacities in manipulating (or at least affecting) their vector's behaviour. In the last few years, *T. cruzi* has indeed been shown to affect the dispersal of *T. dimidiata* (Nouvellet, Ramirez‐Sierra, Dumonteil, & Gourbière, 2011; Ramirez‐Sierra, Herrera‐Aguilar, Gourbière, & Dumonteil, 2010) and *R. prolixus* (Marlière et al., 2015). Even more intriguingly, infected bugs have been shown to bite 45% more often than uninfected individuals, and the time before defecation to be reduced by 30% by infection (Botto‐Mahan, Cattan, & Medel, 2006). While this neat paper did not provide any information on the infecting strain of *T. cruzi*, experiments could presumably be repeated to quantify heterogeneities in the impact of various strains of parasites. The above theory could then be adapted in a data‐driven manner to gain further insights into the contribution of the stercorarian mode of transmission to the low level of virulence of *T. cruzi*.

A second specificity of *T. cruzi* is its ability to infect a broad range of vertebrate species. Both the expression of $\alpha_{h}^{*}$ and the general theory of virulence (Dieckmann, 2002; Otto & Day, 2007) predict shorter lived hosts to lead to greater virulence. As *T. cruzi* is able to infect a vast diversity of vertebrate hosts, its “average host” is likely to be of relatively short life expectancy, so that more virulent strains should outcompete less virulent ones in exploiting such hosts before they die. Noteworthy, these conclusions are based on single host modelling that does not account for a reduction in between strains competition that could be associated with a broad range of host species. Still, the above theory suggests that a larger number of host individuals (*N*_*h*_) would increase *R*_0_, but will not affect the optimal level of virulence, which, again, is mostly because the fitness of all strains is uniformly affected. Hypothetically, a larger number of host species may lead to evolutionary branching and host specialization, or it may yield the evolution of more generalist strategies (Brown, Cornforth, & Mideo, 2012; Gandon, 2004; Leggett, Buckling, Long, & Boots, 2013). However, the analyses of triatomines’ bloodmeal sources have demonstrated that they are generalist haematophagous species feeding on an even broader range of vertebrates than *T. cruzi* is able to infect. Accordingly, the most likely evolutionary scenario is that between‐host species transmission is frequent and, in such conditions, virulence is expected to evolve almost independently of the abundance of the host species, and to be primarily determined by within‐ and between‐hosts constraints on parasite life‐history (Gandon, 2004). To genuinely understand such constraints and ultimately the effects of host biodiversity on the virulence of *T. cruzi* now requires a specific modelling of the pathogen rooted in empirically informed descriptions of its ecological networks.

A third specificity of *T. cruzi* transmission is the longevity and nymphal haematophagy of its triatomine vectors, so that infection rates increase gradually during the entire triatomine lifespan (Buitrago et al., 2010) that typically is one order of magnitude longer than other mosquito or fly vectors (Rascalou et al., 2012). A common wisdom in modelling vectorborne infection is to consider that pathogens have no effect on their vectors (Elliot, Adler, & Sabelis, 2003). Under such assumption (ν = 0), the expected level of *T. cruzi* virulence $\alpha_{h}^{*}$ would not depend on triatomine lifespan. However, significant effects of *T. cruzi* infection have been reported on the behaviour and/or life‐history of various triatomine species, which include *T. infestans* (Schaub, 1988, 1989), *R. prolixus* (Elliot, Rodrigues, Lorenzo, Martins‐Filho, & Guarneri, 2015; Peterson, Bartsch, Lee, & Dobson, 2015), *T. dimidiata* (Ramirez‐Sierra et al., 2010), *P. megistus* (Lima et al., 1992) and *Mepraia spinolai* (Botto‐Mahan, 2009; Botto‐Mahan et al., 2006). This suggests that vectors could be considered as alternative hosts rather than a merely neutral compartment. According to the expression of $\alpha_{h}^{*}$ (with ν ≠ 0), virulence is then expected to decrease with vector life expectancy, so that the unusual lifespan of triatomine may actually be a good candidate hypothesis to explain *T. cruzi* low virulence.

The above predictions are first steps into the development of a theory of *T. cruzi* evolution, and they illustrate how standing eco‐epidemiological models can be informative. Although such optimization of *R*_0_ accounting for potential trade‐off between pathogen's life‐history traits is a natural approach to lay the foundation of the desired theory, it sensu stricto compares the ability of different strains to invade a naive host–vector population (Bull & Ebert, 2008). The outcomes of optimality study often provide satisfactory predictions on longer term evolution were various virulent strains can invade each other in more typical mutation‐substitution dynamics (Cressler, McLeod, Rozins, Van Den Hoogen, & Day, 2015). However, complexes dynamical feedbacks can also emerge when virulent strains are confronted one with another (Dieckmann, 2002) and when host populations experiment recurrent invasions of new parasite variants before equilibrium has been reached (Bull & Ebert, 2008). Understanding the determinants of *T. cruzi* virulence will undoubtedly require combining such approaches, with the ultimate challenges of designing evolution‐proof control strategies to avoid that this ubiquitous pathogen widely spreads across the Americas becomes an even more substantial public health concern.

## 4 CONCLUSION

After the success of international initiatives in reducing the abundance of key domestic vector species in highly endemic areas, new challenges are emerging for the future of Chagas disease control. These new challenges will require a microevolutionary thinking that is slowly growing to assess the evolutionary potential of *T. cruzi* and its triatomine vectors and their adaptive response to control interventions. The eco‐epidemiological relationships that build‐up the selective pressures at work have been assiduously studied over the last century, so that, combined with concepts and modelling inspired from life‐history evolution, a good evolutionary understanding could be rapidly gained. Such basic knowledge will naturally find itself at the heart of future strategies that will undoubtedly target the long‐term sustainability of today's achievements in highly endemic areas and the further reduction in disease incidence in other areas where secondary vectors may be adapting to the human habitat. In such a context, the development of solid eco‐epidemiological studies on a large diversity of triatomine vector species should be encouraged to provide opportunities for comparative analyses that will undoubtedly improve our evolutionary understanding of *T. cruzi* and Chagas disease transmission.

## DATA ARCHIVING STATEMENT

We will not be archiving data because this manuscript does not have associated data.
