# Supplementary material for: Evolutionary ecology of Chagas disease; what do we know and what do we need?
Source: Evol Appl. 2017 Dec 25;11(4):470–87. doi: 10.1111/eva.12582 (PMC5891055; doi:10.1111/eva.12582)
Supplement: Supplementary file 1 [file EVA-11-470-s001.pdf]

Appendix A. Prevalence and incidence of *T. cruzi* infection in Latin America and major international control initiatives.

Prevalence and incidence data from WHO, 2015b; Salvatella, 2007; Silveira et al., 2002; Mancero and Ponce, 2011; WHO, 2005.

International Control Initiative: 1. Amazon initiative. 2. Central America Initiative. 3. Southern Cone Initiative. 4. Andean Initiative.

Country	Prevalence of *T. cruzi* infection (/100 inhabitants)	Incidence of *T. cruzi* vectorial transmission (/100 inhabitants)	Control initiative
Argentine	3.640	0.002	3
Belize	0.33	0.003	2
Bolivia	6.104	0.081	1, 3
Brazil	0.03	0.000084	1,3
Chile	0.669	0	3
Colombia	0.956	0.011	1, 4
Costa Rica	0.169	0.0002	2
Ecuador	1.379	0.014	1, 4
El Salvador	1.297	0.0130	2
Guatemala	1.230	0.009	2
French Guyana, Guyana, and Suriname	0.838	0.018	1
Honduras	0.917	0.011	2
Mexico	0.779	0.005	
Nicaragua	0.522	0.006	2
Panama	0.515	0.004	2
Paraguay	2.130	0.003	3
Peru	0.439	0.007	1, 4
Uruguay	0.237	0	3
Venezuela	0.710	0.0030	1, 4

Appendix B. Review of insecticide resistance in triatomines.

Reference	Sp	C	Province	Ins	Age	Hab	LD_{50}	RR_{50}	M	S*	P**
Pessoa et al., 2015a	TI	Br	Rio Grande Do Sud	D	5d	P	0.733	1.73	100	S	1
	TI	Br	Rio Grande Do Sud	D	5d	P	0.863	2.05	100	S	1
	TI	Br	Rio Grande Do Sud	D	5d	P	1.372	3.26	100	S	1
	TI	Br	Bahia	D	5d	P	0.762	1.74	100	S	1
	TI	Br	Bahia	D	5d	P	0.741	1.76	100	S	1
Pessoa et al., 2015b	TS	Br	Minas Gerais	D	5d	P	0.165	2.5		S	1
	TS	Br	Minas Gerais	D	5d	P	0.18	2.73		S	1
	TS	Br	Minas Gerais	D	5d	P	0.223	3.39		S	1
	TS	Br	Minas Gerais	D	5d	P	0.228	3.47		S	1
	TS	Br	Minas Gerais	D	5d	P	0.235	3.58		S	1
	TS	Br	Minas Gerais	D	5d	P	0.236	3.59		S	1
	TS	Br	Minas Gerais	D	5d	P	0.269	4.8		S	1
	TS	Br	Minas Gerais	D	5d	P	0.285	4.34		S	1
	TS	Br	Minas Gerais	D	5d	P	0.288	4.38		S	1
	TS	Br	Minas Gerais	D	5d	P	0.331	6.388		R	1
	TS	Br	Minas Gerais	D	5d	P	0.354	5.39		R	1
	TS	Br	Minas Gerais	D	5d	P	0.372	5.66		R	1
	TS	Br	Minas Gerais	D	5d	P	0.399	6.6		R	1
	TS	Br	Minas Gerais	D	5d	P	0.465	7.8		R	1
Roca-Acevedo et al., 2013	TI	B	Tarija	D	35d **e**	D	228	1108		HR	
	TI	A	Chaco	D	35d **e**	D	36	173		HR	1
	TI	B	Tarija	D	4d **e**	D	576	1144		HR	
	TI	A	Chaco	D	4d **e**	D	10.63	21		R	
	TI	B	Tarija	D	7d **e**	D	501	1193		HR	1
	TI	A	Chaco	D	7d **e**	D	6	15		R	1
	TI	B	Tarija	D	12d **e**	D	1277	822		HR	
	TI	A	Chaco	D	12d **e**	D	61	39		R	
Pessoa et al., 2015c	TS	Br	Minas Gerais	D	5d	P	0.071	0.84	100	S	1
	TS	Br	Minas Gerais	D	5d	P	0.182	2.8	100	S	1
	TS	Br	Minas Gerais	D	5d	P	0.054	0.84	100	S	1
	TS	Br	Minas Gerais	D	5d	P	0.068	1.05	100	S	1
	TS	Br	Minas Gerais	D	5d	P	0.056	0.86	100	S	1
	TS	Br	Minas Gerais	D	5d	P	0.084	1.31	100	S	1
	TS	Br	Minas Gerais	D	5d	P	0.129	1.99	100	S	1
	TS	Br	Minas Gerais	D	5d	P	0.136	2.1	100	S	1
	TS	Br	Minas Gerais	D	5d	P	0.138	2.13	100	S	1
	TS	Br	Minas Gerais	D	5d	P	0.144	2.22	100	S	1
	TS	Br	Minas Gerais	D	5d	P	0.163	2.51	100	S	1
	TS	Br	Minas Gerais	D	5d	P	0.139	2.15	100	S	1
	TS	Br	Minas Gerais	D	5d	P	0.147	2.27	100	S	1
	TS	Br	Minas Gerais	D	5d	P	0.15	2.32	100	S	1
	TS	Br	Minas Gerais	D	5d	P	0.219	3.38	100	S	1
	TS	Br	Minas Gerais	D	5d	P	0.086	1.33	100	S	1

	TS	Br	Minas Gerais	D	5d	P	0.071	1.09	100	S	1
Germano et al., 2010	TI	A	Salta	D	I (3-5d)	D	31.1	133.1		HR	1
	TI	A	Salta	D	I (3-5d)	D	5.1	39		R	1
	TI	A	Salta	D	I (3-5d)	D	0.02	0.1		S	1
	TI	A	Salta	D	I (3-5d)	D	1.1	8.4		R	1
	TI	A	Catamarca	D	I (3-5d)	D	0.6	4.5		S	1
	TI	A	Santiago del Estero	D	I (3-5d)	D	0.5	3.8		S	1
	TI	A	Santiago del Estero	D	I (3-5d)	D	0.04	0.3		S	1
	TI	A	Chaco	D	I (3-5d)	D	0.5	3.7		S	1
	TI	A	Santa Fé	D	I (3-5d)	D	0.1	0.9		S	1
	TI	B	Tarija	D	I (3-5d)	D	70.1	541.6		HR	1
	TI	B	Tarija	D	I (3-5d)	D	56.7	438		HR	1
	TI	B	Tarija	D	I (3-5d)	D	33.2	299.8		HR	1
	TI	B	Tarija	D	I (3-5d)	D	32	247.4		HR	1
	TI	B	Boqueron	D	I (3-5d)	D	0.5	3.7		S	1
	TI	A	Salta	D+Fi	I (3-5d)	D	4.9	1.9		S	1
	TI	A	Salta	D+Fi	I (3-5d)	D	6.2	3		S	1
	TI	A	Salta	D+Fi	I (3-5d)	D	4.9	2.2		S	1
	TI	A	Salta	D+Fi	I (3-5d)	D	2.3	1.09		S	1
	TI	A	Santiago del Estero	D+Fi	I (3-5d)	D	4.5	2		S	1
	TI	B	Tarija	D+Fi	I (3-5d)	D	0.4	0.19		S	1
	TI	B	Tarija	D+Fi	I (3-5d)	D	205.9	92.7		HR	1
	TI	B	Tarija	D+Fi	I (3-5d)	D	123.1	55.5		HR	1
Depickère et al., 2012	TI	B	La Paz	D	I	S			100	S	1
	TI	B	La Paz	D	I	S			100	S	1
	TI	B	La Paz	D	I	S			100	S	1
	TI	B	La Paz	D	I	S			100	S	1
	TI	B	La Paz	D	I	S			100	S	1
	TI	B	La Paz	D	I	S			100	S	1
	TI	B	La Paz	D	I	S			100	S	1
	TI	B	La Paz	D	I	S			100	S	1
	TI	B	La Paz	D	I	D			100	S	1
	TI	B	Cochabamba	D	I	S			100	S	1
	TI	B	Cochabamba	D	I	S			100	S	1
	TI	B	Cochabamba	D	I	D			100	S	1
	TI	B	Cochabamba	D	I	S			96		1
	TI	B	Potosi	D	I	S			79	R	1
	TI	B	Santa Cruz	D	I	P			88		1
	TI	B	Santa Cruz	D	I	D	0.91	3.8	94	S	1
	TI	B	Santa Cruz	D	I	D	1.99	8.29	74	R	1
	TI	B	Santa Cruz	D	I	D	1.98	8.25	80	R	1
	TI	B	Santa Cruz	D	I	D			90		1
	TI	B	Santa Cruz	D	I	D	196.38	828	0	HR	1
Lardeux et al., 2010	TI	B	Chuquisaca	D	I	D	277	735	6	HR	1
	TI	B	Chuquisaca	D	I	D			45	R	1
	TI	B	Chuquisaca	D	I	D			66	R	1
	TI	B	Chuquisaca	D	I	P			67	R	1

	TI	B	Chuquisaca	D	I	D	6.9		88		1
	TI	B	Chuquisaca	D	I	P	5.2		98	S	1
	TI	B	Chuquisaca	D	I	D			40	R	1
	TI	B	Chuquisaca	D	I	D			40	R	1
	TI	B	Chuquisaca	D	I	D	5.9		100	S	1
	TI	B	Chuquisaca	D	I	D	8.5		72	R	1
	TI	B	Chuquisaca	D	I	D	33		17	HR	1
	TI	B	Chuquisaca	D	I	P	11		82		1
	TI	B	Chuquisaca	D	I	D	21		48	R	1
	TI	B	Chuquisaca	D	I	P	14		58	R	1
	TI	B	Chuquisaca	D	I	D			48	R	1
	TI	B	Chuquisaca	D	I	D	5		100	S	1
	TI	B	Chuquisaca	D	I	D	5		91		1
	TI	B	Chuquisaca	D	I	D	323	936	0	HR	1
	TI	B	Chuquisaca	D	I	D			5	HR	1
	TI	B	Chuquisaca	D	I	D	230		0	HR	1
	TI	B	Chuquisaca	D	I	P	142	307	3	HR	1
	TI	B	Chuquisaca	D	I	D	13		77	R	1
	TI	B	Cochabamba	D	I	D			73	R	1
	TI	B	Cochabamba	D	I	D	14		67	R	1
	TI	B	Cochabamba	D	I	D			92		1
	TI	B	La Paz	D	I	D			100	S	1
	TI	B	La Paz	D	I	D	5.8		100	S	1
	TI	B	La Paz	D	I	S			100	S	1
	TI	B	La Paz	D	I	D	9.5		95		1
	TI	B	La Paz	D	I	D			100	S	
	TI	B	La Paz	D	I	D			0	HR	1
	TI	B	La Paz	D	I	P			76	R	1
	TI	B	La Paz	D	I	D	3.7		92		1
	TI	B	La Paz	D	I	D			63	R	1
	TI	B	Tarija	D	I	D	165	424	31	HR	1
	TI	B	Tarija	D	I	D	204	924	1	HR	1
	TI	B	Tarija	D	I	D			6	HR	1
	TI	B	Tarija	D	I	D	131	530	15	HR	1
	TI	B	Tarija	D	I	D			8	HR	1
	TI	B	Tarija	D	I	D			33	R	1
	TI	B	Tarija	D	I	D	96	274	17	HR	1
	TI	B	Tarija	D	I	D	315	1157	9	HR	1
	TI	B	Tarija	D	I	D	30	189	43	HR	1
	TI	B	Tarija	D	I	D	491	1170	5	HR	1
	TI	B	Tarija	D	I	D	148		38	R	1
	TI	B	Tarija	D	I	D	214		3	HR	1
	TI	B	Tarija	D	I	D	33	405	44	HR	1
	TI	B	Chuquisaca	D+PBO	I	D	3.01	12.4		R	1
	TI	B	Tarija	D+PBO	I	D	37.52	153		HR	1
	TI	B	Tarija	D+PBO	I	D	33.79	144		HR	1
	TI	B	Tarija	D+PBO	I	P	9.07	38		R	1
	TI	B	Tarija	D+PBO	I	D	33.82	139		HR	1
	TI	B	Tarija	D+PBO	I	D	39.17	147		HR	1

	TI	B	Tarija	D+PBO	I	D	4.32	18		R	1
	TI	B	Chuquisaca	Be	I	D			100	S	1
	TI	B	Chuquisaca	Be	I	P			100	S	1
	TI	B	Chuquisaca	Be	I	D			96		1
	TI	B	Chuquisaca	Be	I	D			100	S	1
	TI	B	Chuquisaca	Be	I	D			88		1
	TI	B	Chuquisaca	Be	I	D			81		1
	TI	B	Chuquisaca	Be	I	D			100	S	1
	TI	B	Chuquisaca	Be	I	D			100	S	1
	TI	B	Cochabamba	Be	I	D			93		1
	TI	B	Cochabamba	Be	I	D			100	S	1
	TI	B	La Paz	Be	I	D	17.67	1.6	100	S	1
	TI	B	Tarija	Be	I	D	19.56	1.8	98	S	1
	TI	B	Tarija	Be	I	D	15.18	1.4	100	S	1
	TI	B	Tarija	Be	I	D	17.73	1.6	95	S	1
	TI	B	Tarija	Be	I	D			91		1
	TI	B	Tarija	Be	I	D			100	S	1
	TI	B	Tarija	Be	I	D			95		1
	TI	B	Tarija	Be	I	D			100	S	1
	TI	B	Tarija	Be	I	D			97		1
	TI	B	Tarija	Be	I	D			94		1
	TI	B	Tarija	Be	I	D	18.8	1.7	100	S	1
	TI	B	Tarija	Be	I	D			100	S	1
	TI	B	Tarija	Be	I	D			100	S	1
	TI	B	Tarija	Be	I	D	20.82	1.7	94	S	1
	TI	B	Tarija	Be	I	D			98	S	1
	TI	B	Tarija	Be	I	D	20.7	1.8	100	S	1
	TI	B	Tarija	Be	I	D	16.52	1.5	100	S	1
	TI	B	Cochabamba	Ma	I	D			91		1
	TI	B	La Paz	Ma	I	D			100	S	1
	TI	B	Tarija	Ma	I	D			99	S	1
	TI	B	Tarija	Ma	I	D			100	S	1
	TI	B	Tarija	Ma	I	D			100	S	1
	TI	B	Tarija	Ma	I	D			100	S	1
	TI	B	Tarija	Ma	I	D	38.81	2.2	96	S	1
	TI	B	Tarija	Ma	I	D	31.14	1.8	100	S	1
	TI	B	Tarija	Ma	I	D			95		1
	TI	B	Tarija	Ma	I	D			100	S	1
	TI	B	Tarija	Ma	I	D			99	S	1
	TI	B	Tarija	Ma	I	D			100	S	1
	TI	B	Tarija	Ma	I	D			100	S	1
	TI	B	Tarija	Ma	I	D			100	S	1
	TI	B	Tarija	Ma	I	D			100	S	1
	TI	B	Tarija	Ma	I	D			100	S	1
	TI	B	Tarija	Ma	I	D			100	S	1
Bustamante Gomez et al., 2014	TI	B	Santa Cruz	D	I	S	0.26	0.62	100	S	1
	TI	B	Santa Cruz	D	I	S	0.39	0.93	100	S	1
	TI	B	Santa Cruz	D	I	S	0.48	1.16	100	S	1
	TI	B	Cochabamba	D	I	S	1.78	4.24	96	S	1

	TI	B	Cochabamba	D	I	S	1.22	2.9	100	S	1
	TI	B	Cochabamba	D	I	D+P	3.52	8.49	62	R	1
	TI	B	Santa Cruz	D	I	D+P	1.75	4.21	93	S	1
	TI	B	Santa Cruz	D	I	D+P	2.09	5.04	93	R	1
	TI	B	Tarija	D	I	P	54.23	129.12	0	HR	1
Vassena et al., 2000	TI	Br	Rio Grande Do Sud	D	I (3d)	D	0.7	7		R	1
	TI	Br	Rio Grande Do Sud	Bcp	I (3d)	D	0.24	0.92		S	1
	TI	Br	Rio Grande Do Sud	BC	I (3d)	D	0.36	3.6		S	1
	TI	Br	Rio Grande Do Sud	LC	I (3d)	D	0.28	1.75		S	1
	TI	Br	Rio Grande Do Sud	Cy	I (3d)	D	1.34	3.35		S	1
	TI	Br	Rio Grande Do Sud	D+PBO	I (3d)	D	0.1		100	S	1
	RP	V	Cardabobo	D	I (3d)	D	0.114	11.4		R	1
	RP	V	Cardabobo	Bcp	I (3d)	D	0.28	7.9		R	1
	RP	V	Cardabobo	BC	I (3d)	D	0.142	6.8		R	1
	RP	V	Cardabobo	LC	I (3d)	D	0.091	4.5		S	1
	RP	V	Cardabobo	Di	I (3d)	D	23.34	3		S	1
	RP	V	Cardabobo	Cy	I (3d)	D	0.99	12.4		R	1
	RP	V	Cardabobo	D+PBO	I (3d)	D	0.012		100	S	1
Sonoda et al., 2010	TB	Br	Ceara	D	I (5d)	D	0.33	1.74		S	1
	TB	Br	Ceara	D	I (5d)	D	0.22	2.83		S	1
	TB	Br	Ceara	D	I (5d)	D	0.34	1.79		S	1
	TB	Br	Ceara	D	I (5d)	D	0.3	1.58		S	1
	TB	Br	Ceara	D	I (5d)	P	0.24	1.26		S	1
	TB	Br	Ceara	D	I (5d)	P	0.33	1.74		S	1
	TB	Br	Ceara	D	I (5d)	P	0.3	1.58		S	1
	TB	Br	Ceara	D	I (5d)	S	0.19	1		S	1
Pires et al., 2000	TI	Br	Minas Gerais	D	V	D			23.3	HR	1
	TI	B	Cochabamba	D	V	P			16.6	HR	1
Germano et al., 2010b	TI	A	Salta	D	I (3-5d)		0.07	0.55		S	1
	TI	A	Salta	D	I (3-5d)		60.87	856.84		HR	1
Germano et Picollo, 2014	TI	A	Salta	D	I (7d)	D+P	38.8	343.2		HR	1
	TI	A	Santiago del Estero	D	I (7d)	D+P	0.11		50	R	1
Germano et al., 2013	TI	A	Salta	D	I (5-7d)	P	4.87	37.51	30	R	1
	TI	A	Salta	D	I (5-7d)	P	2.59	19.9	62	R	1
	TI	A	Salta	D	I (5-7d)	P	2.16	16.71	45	R	1
	TI	A	Chaco	D	I (5-7d)	D	122.67	946.11	0	HR	1
	TI	A	Chaco	D	I (5-7d)	D			0	HR	1
	TI	A	Chaco	D	I (5-7d)	D	0.15	1.2	76	S	1
	TI	A	Chaco	D	I (5-7d)	P	68.78	529.09	0	HR	1
	TI	A	Chaco	D	I (5-7d)	P			0	HR	1
	TI	A	Chaco	D	I (5-7d)	P	191.89	1417.7	3	HR	1
	TI	A	Chaco	D	I (5-7d)	P	1.11	8.62	60	R	1
	TI	A	Chaco	D	I (5-7d)	P	0.35	2.74	100	S	1
	TI	A	Chaco	D	I (5-7d)	P	0.14	1.09	91	S	1
	TI	A	Chaco	D	I (5-7d)	P	0.13	0.97	70	S	1

	TI	A	Chaco	D	I (5-7d)	P	0.16	1.23	81	S	1
	TI	A	Chaco	D	I (5-7d)	P			100	S	1
	TI	A	Chaco	D	I (5-7d)	P	0.12	0.9	93	S	1
	TI	A	Chaco	D	I (5-7d)	P	0.12	0.9	97	S	1
Fabro et al., 2013	TI	A	Salta	D	I (5-7d)	D	4.7	35.7		R	1
González et al., 2004	TI	A	Mendoza	D	I (3d)		0.375	3.771		S	1
	TI	A	San Luis	D	I (3d)		1.999	2.2		S	1
	TI	A	Catamaraca	D	I (3d)		0.266	2.679		S	1
	TI	A	Salta	D	I (3d)	D+P	0.78	7			
Fronza et al., 2016	TI	A	Mendoza	D	I (5-7d)	D+P			94		1
	TI	A	Mendoza	D	I (5-7d)	D+P			100	S	1
	TI	A	Mendoza	D	I (5-7d)	D+P			100	S	1
	TI	A	Mendoza	D	I (5-7d)	D+P			100	S	1
	TI	A	Mendoza	D	I (5-7d)	D+P			100	S	1
	TI	A	Mendoza	D	I (5-7d)	D+P			100	S	1
	TI	A	Mendoza	D	I (5-7d)	D+P			95		1
	TI	A	San Juan	D	I (5-7d)	D+P			90		1
	TI	A	San Juan	D	I (5-7d)	D+P			81		1
	TI	A	San Juan	D	I (5-7d)	D+P			100	S	1
	TI	A	Santiago del Estero	D	I (5-7d)	D+P			96		1
	TI	A	Santiago del Estero	D	I (5-7d)	D+P			82		1
	TI	A	Santiago del Estero	D	I (5-7d)	D+P			91		1
	TI	A	Santiago del Estero	D	I (5-7d)	D+P			93		1
	TI	A	Santiago del Estero	D	I (5-7d)	D+P			100	S	1
	TI	A	Santiago del Estero	D	I (5-7d)	D+P			100	S	1
	TI	A	Santiago del Estero	D	I (5-7d)	D+P			81		1
	TI	A	Santiago del Estero	D	I (5-7d)	D+P			100	S	1
	TI	A	Santiago del Estero	D	I (5-7d)	D+P			89		1
	TI	A	Santiago del Estero	D	I (5-7d)	D+P			95		1
	TI	A	Santiago del Estero	D	I (5-7d)	D+P			93		1
	TI	A	Tucuman	D	I (5-7d)	D+P			80		1
	TI	A	Tucuman	D	I (5-7d)	D+P			94		1
	TI	A	Tucuman	D	I (5-7d)	D+P			96		1
	TI	A	Tucuman	D	I (5-7d)	D+P			80		1
	TI	A	Tucuman	D	I (5-7d)	D+P			80		1
	TI	A	Tucuman	D	I (5-7d)	D+P			100	S	1
	TI	A	Chaco	D	I (5-7d)	D+P	>200	>1000	3	HR	1
	TI	A	Chaco	D+Fe		D+P			100	S	1
	TI	A	Chaco	D	I (5-7d)	D+P			13	HR	1
	TI	A	Chaco	D	I (5-7d)	D+P	115.2	500	13	HR	1
	TI	A	Chaco	D+Fe		D+P			100	S	1
	TI	A	Chaco	D	I (5-7d)	D+P	>200	>1000	2	HR	1

	TI	A	Chaco	D		D+P			100	S	1
	TI	A	Chaco	D	I (5-7d)	D+P	>200	>1000	0	HR	1
	TI	A	Chaco	D+Fe		D+P			100	S	1
	TI	A	Chaco	D	I (5-7d)	D+P	>200	>1000	0	HR	1
	TI	A	Chaco	D+Fe		D+P			100	S	1
	TI	A	Chaco	D	I (5-7d)	D+P	0.87	4.72	90	S	1
	TI	A	Chaco	D+Fe		D+P			96.7		1
	TI	A	Chaco	D	I (5-7d)	D+P	1.08	5.68	93	R	1
	TI	A	Chaco	D		D+P			93.3		1
	TI	A	Chaco	D	I (5-7d)	D+P	0.61	3.29	100	S	1
	TI	A	Chaco	D+Fe		D+P			100	S	1
	TI	A	Chaco	D	I (5-7d)	D+P	0.96	5.21	83	R	1
	TI	A	Chaco	D+Fe		D+P			100	S	1
	TI	A	Chaco	D	I (5-7d)	D+P	0.74	4.03	77	S	1
	TI	A	Chaco	D+Fe		D+P			100	S	1
	TI	A	Chaco	D	I (5-7d)	D+P	0.77	4.18	77	S	1
	TI	A	Chaco	D+Fe		D+P			100	S	1
	TI	A	Chaco	D	I (5-7d)	D+P	0.67	3.97	92	S	1
	TI	A	Chaco	D+Fe		D+P			90		1
	TI	A	Chaco	D	I (5-7d)	D+P	0.56	3.06	84	S	1
	TI	A	Chaco	D		D+P			100	S	1
	TI	A	Chaco	D	I (5-7d)	D+P	0.04	0.19	88	S	1
	TI	A	Chaco	D	I (5-7d)	D+P	0.04	0.2	100	S	1
	TI	A	Chaco	D	I (5-7d)	D+P	0.04	0.21	82	S	1
	TI	A	Chaco	D	I (5-7d)	D+P	0.37	2.02	100	S	1
	TI	A	Chaco	D	I (5-7d)	D+P	0.44	2.38	92	S	1
	TI	A	Chaco	D	I (5-7d)	D+P			96		1
	TI	A	Chaco	D	I (5-7d)	D+P			100	S	1
	TI	A	Chaco	D	I (5-7d)	D+P			100	S	1
	TI	A	Chaco	D	I (5-7d)	D+P			97		1
	TI	A	Chaco	D	I (5-7d)	D+P			94		1
	TI	A	Chaco		I (5-7d)	D+P			100	S	1
	TI	A	Chaco	Fe	I (5-7d)	D+P			100	S	1
	TI	A	Chaco	Fe	I (5-7d)	D+P			100	S	1
	TI	A	Chaco	Fe	I (5-7d)	D+P			100	S	1
	TI	A	Chaco	Fe	I (5-7d)	D+P			100	S	1
	TI	A	Chaco	Fe	I (5-7d)	D+P			96.7		1
	TI	A	Chaco	Fe	I (5-7d)	D+P			93.3		1
	TI	A	Chaco	Fe	I (5-7d)	D+P			100	S	1
	TI	A	Chaco	Fe	I (5-7d)	D+P			100	S	1
	TI	A	Chaco	Fe	I (5-7d)	D+P			100	S	1
	TI	A	Chaco	Fe	I (5-7d)	D+P			100	S	1
	TI	A	Chaco	Fe	I (5-7d)	D+P			90		1
	TI	A	Chaco	Fe	I (5-7d)	D+P			100	S	1
	TI	A	Chaco	D	10d **e**	D+P			6.5	HR	1
	TI	A	Chaco	D	10d **e**	D+P			0	HR	1
	TI	A	Chaco	D	10d **e**	D+P			20	HR	1
	TI	A	Chaco	D	10d **e**	D+P			2	HR	1
	TI	A	Chaco	D	10d **e**	D+P			0	HR	1

	TI	A	Chaco	D	10d **e**	D+P			93		1
	TI	A	Chaco	D	10d **e**	D+P			100	S	1
	TI	A	Chaco	D	10d **e**	D+P			70	R	1
	TI	A	Chaco	D	10d **e**	D+P			100	S	1
	TI	A	Chaco	D	10d **e**	D+P			87		1
	TI	A	Chaco	D	10d **e**	D+P			25	HR	1
Picollo et al., 2005	TI	A	Salta	D	I (3d)	D	12.8	99		HR	1
	TI	A	Salta	D	I (3d)	D	11.3	86.9		HR	1
	TI	A	Salta	D	I (3d)	D	6.5	50.5		HR	1
	TI	A	Salta	D	I (3d)	D	31.1	133.1		HR	1
	TI	A	Salta	Bcp	I (3d)	D	106.5	451.2		HR	1
	TI	A	Salta	Bcp	I (3d)	D	63.7	270.1		HR	1
	TI	A	Salta	Bcp	I (3d)	D	62.2	263.5		HR	1
	TI	A	Salta	Bcp	I (3d)	D	72.8	308.4		HR	1
	TI	A	Salta	BC	I (3d)	D	27.1	171.7		HR	1
	TI	A	Salta	BC	I (3d)	D	23.4	148.4		HR	1
	TI	A	Salta	BC	I (3d)	D	9.5	60.3		HR	1
	TI	A	Salta	BC	I (3d)	D	105.1	667.6		HR	1
	TI	A	Salta	Fe	I (3d)	D	27	1.29		S	1
	TI	A	Salta	LC	I (3d)	D	53.3	288.7		HR	1
	TI	A	Salta	Fe	I (3d)	D	25.7	1.54		S	1
	TI	A	Salta	LC	I (3d)	D	26.2	141.7		HR	1
	TI	A	Salta	Fe	I (3d)	D	36.1	1.78		S	1
	TI	A	Salta	LC	I (3d)	D	11.9	64.5		HR	1
	TI	A	Salta	Fe	I (3d)	D	23.4	1.39		S	1
	TI	A	Salta	LC	I (3d)	D	19.6	106.2		HR	1
	TI	A	Salta				19.5	101.1		HR	1
	TI	A	Salta				33.8	133.9		HR	1
Santo-Orihuela et al., 2013	TI	B	Cochabamba	Fe	I	S	20.2	1.87		S	1
	TI	B	Potosi	Fe	I	S	14.6	1.35		S	1
	TI	B	Tarija	Fe	I	D	17.4	1.61		S	1
	TI	B	Cochabamba	Fe	I	S	26.8	2.47		S	1
Sierra et al., 2016	TI	A	Chaco	D		D		2.08		S	1
	TI	A	Chaco	Fe		D		1.45		S	1
	TI	A	Chaco	D		D		5.51		R	1
	TI	A	Chaco	Fe		D		1.2		S	1
	TI	A	Chaco	D		D		>2000		HR	1
	TI	A	Chaco	Fe		D		0.84		S	1
Germano et al., 2012	TI	A	Salta	D	I (5-7d)	D+P	6.4	32.5		R	1
	TI	A	Chaco	Fi	I (5-7d)	D+P	33.6	2.6		S	1
	TI	B	Cochabamba	Fe	I (5-7d)	D+P	30.2	2.6		S	1
	TI	A	Salta	D	I (5-7d)	D+P	35.8	173.8		HR	1
	TI	A	Chaco	Fi	I (5-7d)	D+P	155.1	12.4		R	1
	TI	B	Cochabamba	Fe	I (5-7d)	D+P	19.4	1.6		S	1
	TI	A	Salta	D	I (5-7d)	D+P	2.3	17.4		R	1
	TI	A	Chaco	Fi	I (5-7d)	D+P	859.8	66.8		HR	1
	TI	B	Cochabamba	Fe	I (5-7d)	D+P	15.3	1.3		S	1
	TI	A	Salta	D	12d **e**	D+P	44.5	28.6		R	1
	TI	A	Chaco	D	12d **e**	D+P	60.6	39.1		R	1

	TI	B	Cochabamba	D	12d **e**	D+P	13	8.4		R	1
Toloza et al., 2008	TI	A	Salta	LC	11-12d **e**		44.1	14.2		R	1
	TI	B	Cochabamba	LC	11-12d **e**		6	2.1		S	1
	TI	B	Chuquisaca	LC	11-12d **e**		8.1	2.5		S	1
	TI	A	Salta	Fi	11-12d **e**		4922	1.3		S	1
	TI	B	Cochabamba	Fi	11-12d **e**		2736	0.7		S	1
	TI	B	Chuquisaca	Fi	11-12d **e**		7438	2		S	1
	TI	A	Salta	Fe	11-12d **e**		80	1		S	1
	TI	B	Cochabamba	Fe	11-12d **e**		32	0.4		S	1
	TI	B	Chuquisaca	Fe	11-12d **e**		63	0.8		S	1
	TI	A	Salta	LC	I (5-7d)		19.6	106.2		HR	1
	TI	B	Cochabamba	LC	I (5-7d)		0.53	2.9		S	1
	TI	B	Chuquisaca	LC	I (5-7d)		0.51	2.8		S	1
	TI	A	Salta	Fi	I (5-7d)		4.96	1.9		S	1
	TI	B	Cochabamba	Fi	I (5-7d)		859.8	386.8		HR	1
	TI	B	Chuquisaca	Fi	I (5-7d)		1301.2	585.5		HR	1
	TI	A	Salta	Fe	I (5-7d)		23.4	1.39		S	1
	TI	B	Cochabamba	Fe	I (5-7d)		15.32	0.72		S	1
	TI	B	Chuquisaca	Fe	I (5-7d)		21.89	1.03		S	1
Yon et al., 2001	TI	P	Arequipa	C	Adults				100	S	1
	TI	P	Arequipa	C	V				100	S	1
	TI	P	Arequipa	BC	Adults				100	S	1
	TI	P	Arequipa	BC	V				100	S	1
	TI	P	Arequipa	Cy	Adults				100	S	1
	TI	P	Arequipa	Cy	V				100	S	1
	TI	P	Arequipa	AC	Adults				100	S	1
	TI	P	Arequipa	AC	V				100	S	1
	TI	P	Arequipa	D	Adults				100	S	1
	TI	P	Arequipa	D	V				100	S	1
	TI	P	Arequipa	D	Adults				100	S	1
	TI	P	Arequipa	D	V				100	S	1
	TI	P	Arequipa	BC	Adults				66	R	1
	TI	P	Arequipa	BC	V				57	R	1
	TI	P	Arequipa	AC	Adults				87		1
	TI	P	Arequipa	AC	V				87		1
	TI	P	Arequipa	D	Adults				30	R	1
	PH	P	Cutervo	C	Adults				97		1
	PH	P	Cutervo	C	V				97		1
	PH	P	Cutervo	AC	Adults				100	S	1
	PH	P	Cutervo	AC	V				56	R	1
	PH	P	Jaén	AC	Adults				100	S	1
	PH	P	Cutervo	AC	V				100	S	1
	PH	P	Jaén	D	Adults				100	S	1
	PH	P	Jaén	D	V				100	S	1
	PH	P	San Ignacio	AC	Adults				100	S	1
	PH	P	San Ignacio	AC	V				50	R	1
	PH	P	Cutervo	D	Adults				100	S	1
	PH	P	Cutervo	D	V				100	S	1
Roca-Acevedo et	TI	B	Cochabamba		I	D	0,86			R	1

al., 2011	TI	B	Cochabamba		I	S	0,66			R	1
	TI	B	Cochabamba		I	S	0,13			R	1
	TI	B	Cochabamba		I	S	0,37			R	1
	TI	B	Potosi		I	S	0,43			S	1
	TI	B	Cochabamba	Fi	I	D	152.5			HR	1
	TI	B	Cochabamba	Fi	I	S	21,56			R	1
	TI	B	Cochabamba	Fi	I	S	44,24			R	1
	TI	B	Cochabamba	Fi	I	S	0.43			R	1
	TI	B	Potosi	Fi	I	S	3,98			R	1
Germano et al., 2014	TI	A	Chaco	D	I (5-7d)	D	30.32	233.42		HR	1
	TI	A	Chaco	Fe	I (5-7d)	D	21.38	21.38		S	

Symbols

Sp : Species. TI: *T. infestans,* PH: *P. herreri*, RP: *R. prolixus*, TB: *T. brasiliensis*, TS: *T. sordida*

C: Country. A: Argentina. B: Bolivia, Br: Brazil, P: Peru

Ins: Insecticide. AC:Alpha-cypermethrin, Be: Bendiocarb, BC: Beta-cyfluthrin, Bpc: Beta-cypermethrin, C: Cyfluthrin, Cy: Cypermethrin, D: Deltameltrhin, Di: Dieldrin, Fe: Fenitrohion, Fi: Fipronil, LC: Lambda-cyhalothrin, Ma: Malathion, PBO: Piperonyl butoxide.

Hab: Habitat. D: Domestic, P: Peridomestic, S: Sylvatic.

Age. d: day, I : first instar nymphs; V: five instar nymphs, e:eggs

LD: Lethal Dose, RR: Resistance Ratio. M: Mortality

S: Status. R: Resistant, HR: High Resistant. S: Susceptible.

*Population status is HR if RR_{50}>50 or M<30, R if (RR50≤50 & RR50>5) or (30<M<80), S if (RR50≤5 or M>96).

P: Population

**’1’ stands for populations that were consider as “one” population in the data analysys

References:

Bustamante Gomez, M., D’Avila, G. C. P., Orellana, A. L. G., Cortez, M. R., Rosa, A. C. L., Noireau, F., & Diotaiuti, L. G. (2014). Susceptibility to deltamethrin of wild and domestic populations of *Triatoma infestans* of the Gran Chaco and the Inter-Andean Valleys of Bolivia. *Parasites & Vectors*, *7*(1), 497. https://doi.org/10.1186/s13071-014-0497-3

Depickère, S., Buitrago, R., Siñani, E., Baune, M., Monje, M., Lopez, R., … Brenière, S. F. (2012). Susceptibility and resistance to deltamethrin of wild and domestic populations of *Triatoma infestans* (Reduviidae: Triatominae) in Bolivia: new discoveries. *Memórias Do Instituto Oswaldo Cruz*, *107*(8), 1042–1047.

Fabro, J., Sterkel, M., Capriotti, N., Mougabure-Cueto, G., Germano, M., Rivera-Pomar, R., & Ons, S. (2012). Identification of a point mutation associated with pyrethroid resistance in the para-type sodium channel of *Triatoma infestans*, a vector of Chagas' disease. *Infection, Genetics and Evolution*, *12*(2), 487–491. https://doi.org/10.1016/j.meegid.2011.12.006

Fronza, G., Toloza, A. C., Picollo, M. I., Spillmann, C., & Mougabure-Cueto, G. A. (2016). Geographical Variation of Deltamethrin Susceptibility of *Triatoma infestans* (Hemiptera: Reduviidae) in Argentina With Emphasis on a Resistant Focus in the Gran Chaco. *Journal of Medical Entomology*, *53*(4), 880–887. https://doi.org/10.1093/jme/tjw056

Germano, M. D., Acevedo, G. R., Cueto, G. A. M., Toloza, A. C., Vassena, C. V., & Picollo, M. I. (2010a). New Findings of Insecticide Resistance in *Triatoma infestans* (Heteroptera: Reduviidae) From the Gran Chaco. *Journal of Medical Entomology*, *47*(6), 1077–1081. https://doi.org/10.1603/ME10069

Germano, M. D., Vassena, C. V., & Picollo, M. I. (2010b). Autosomal inheritance of deltamethrin resistance in field populations of *Triatoma infestans* (Heteroptera: Reduviidae) from Argentina. *Pest Management Science*, *66*(7), 705–708. https://doi.org/10.1002/ps.1931

Germano, M. D., Santo-Orihuela, P., Roca-Acevedo, G., Toloza, A. C., Vassena, C., Picollo, M. I., & Mougabure-Cueto, G. (2012). Scientific Evidence of Three Different Insecticide-Resistant Profiles in *Triatoma infestans* (Hemiptera: Reduviidae) Populations From Argentina and Bolivia. *Journal of Medical Entomology*, *49*(6), 1355–1360. https://doi.org/10.1603/ME12070

Germano, M. D., Picollo, M. I., & Mougabure-Cueto, G. A. (2013). Microgeographical study of insecticide resistance in *Triatoma infestans* from Argentina. *Acta Tropica*, *128*(3), 561–565. https://doi.org/10.1016/j.actatropica.2013.08.007

Germano, M. D., & Picollo, M. I. (2014). Reproductive and developmental costs of deltamethrin resistance in the Chagas disease vector *Triatoma infestans*. *Journal of Vector Ecology*, *40*(1), 59–65.

Germano, M. D., Picollo, M. I., Spillmann, C., & Mougabure-Cueto, G. (2014). Fenitrothion: an alternative insecticide for the control of deltamethrin-resistant populations of *Triatoma infestans* in northern Argentina: Deltamethrin-resistant *T . infestans* in Argentina. *Medical and Veterinary Entomology*, *28*(1), 21–25. https://doi.org/10.1111/mve.12014

González Audino, P., Vassena, C., Barrios, S., Zerba, E., & Picollo, M. I. (2004). Role of enhanced detoxication in a deltamethrin-resistant population of *Triatoma infestans* (Hemiptera, Reduviidae) from Argentina. *Memorias Do Instituto Oswaldo Cruz*, *99*(3), 335–339.

Lardeux, F., Depickère, S., Duchon, S., & Chavez, T. (2010). Insecticide resistance of *Triatoma infestans* (Hemiptera, Reduviidae) vector of Chagas disease in Bolivia: Insecticide resistance of *Triatoma infestans*. *Tropical Medicine & International Health*, no–no. https://doi.org/10.1111/j.1365-3156.2010.02573.x

Pessoa, G. C. D., Rosa, A. C. L., Bedin, C., Wilhelms, T., Mello, F. de, Coutinho, H. S., … Diotaiuti, L. (2015a). Susceptibility characterization of residual Brazilian populations of *Triatoma infestans* Klug, 1834 (Hemiptera: Reduviidae) to deltamethrin pyrethroid. *Revista Da Sociedade Brasileira de Medicina Tropical*, *48*(2), 157–161. https://doi.org/10.1590/0037-8682-0011-2015

Pessoa, G. C. Dá., Obara, M. T., Rezende, J. G., de Mello, B. V., Ferraz, M. L., & Diotaiuti, L. (2015b). Deltamethrin toxicological profile of peridomestic *Triatoma sordida* in the North of Minas Gerais, Brazil. *Parasites & Vectors*, *8*(1). https://doi.org/10.1186/s13071-015-0873-7

Pessoa, G. C. Dá., Santos, T. R. M. dos, Salazar, G. C., Dias, L. S., Mello, B. V. de, Ferraz, M. L., & Diotaiuti, L. (2015c). Variability of susceptibility to deltamethrin in peridomestic *Triatoma sordida* from Triângulo Mineiro, State of Minas Gerais, Brazil. *Revista Da Sociedade Brasileira de Medicina Tropical*, *48*(4), 417–421. https://doi.org/10.1590/0037-8682-0047-2015

Picollo, M. I., Vassena, C., Orihuela, P. S., Barrios, S., Zaidemberg, M., & Zerba, E. (2005). High resistance to pyrethroid insecticides associated with ineffective field treatments in *Triatoma infestans* (Hemiptera: Reduviidae) from Northern Argentina. *Journal of Medical Entomology*, *42*(4), 637–642.

Pires, H. H. R., Barbosa, S. E., & Diotaiuti, L. (2000). Comparative developmental and susceptibility to insecticide of Bolivian and Brazilian populations of *Triatoma infestans*. *Memórias Do Instituto Oswaldo Cruz*, *95*(6), 883–888.

Roca-Acevedo, G. , Cueto, G. M., Germano, M., Orihuela, P. S., Cortez, M. R., Noireau, F., … Vassena, C. (2011). Susceptibility of Sylvatic *Triatoma infestans* From Andeans Valleys of Bolivia to Deltamethrin and Fipronil. *Journal of Medical Entomology*, *48*(4), 828–835. https://doi.org/10.1603/ME10208

Roca-Acevedo, G., Picollo, M. I., & Santo-Orihuela, P. (2013). Expression of Insecticide Resistance in Immature Life Stages of *Triatoma infestans* (Hemiptera: Reduviidae). *Journal of Medical Entomology*, *50*(4), 816–818. https://doi.org/10.1603/ME12116

Santo-Orihuela, P. L., Carvajal, G., Picollo, M. I., & Vassena, C. V. (2013). Toxicological and biochemical analysis of the susceptibility of sylvatic *Triatoma infestans* from the Andean Valley of Bolivia to organophosphate insecticide. *Memórias Do Instituto Oswaldo Cruz*, *108*(6), 790–795. https://doi.org/10.1590/0074-0276108062013017

Sierra, I., Capriotti, N., Fronza, G., Mougabure-Cueto, G., & Ons, S. (2016). Kdr mutations in *Triatoma infestans* from the Gran Chaco are distributed in two differentiated foci: Implications for pyrethroid resistance management. *Acta Tropica*, *158*, 208–213. https://doi.org/10.1016/j.actatropica.2016.03.014

Sonoda, I. V., Dias, L. S., Bezerra, C. M., Dias, J. C. P., Romanha, A. J., & Diotaiuti, L. (2010). Susceptibility of Triatoma brasiliensis from state of Ceará, Northeastern Brazil, to the pyrethroid deltamethrin. *Memórias Do Instituto Oswaldo Cruz*, *105*(3), 348–352.

Toloza, A. C., Germano, M., Cueto, G. M., Vassena, C., Zerba, E., & Picollo, M. I. (2008). Differential Patterns of Insecticide Resistance in Eggs and First Instars of Triatoma infestans (Hemiptera: Reduviidae) from Argentina and Bolivia. *Journal of Medical Entomology*, *45*(3), 421–426. https://doi.org/10.1603/0022-2585(2008)45[421:DPOIRI]2.0.CO;2

Vassena, C. V., Picollo, M. I., & Zerba, E. N. (2000). Insecticide resistance in Brazilian *Triatoma infestans* and Venezuelan *Rhodnius prolixus*. *Medical and Veterinary Entomology*, *14*(1), 51–55. https://doi.org/10.1046/j.1365-2915.2000.00203.x

Yon, C., Balta, R., García, N., Troyes, M., Cumpa, H., & Valdivia, A. (2004). Susceptibilidad y resistencia de *Triatoma infestans* y *Panstrongylus herreri* a los insecticidas piretroides, Perú 2001. *Revista Peruana de Medicina Experimental Y Salud Publica*, *21*(3), 179–182.

Appendix C: Review of R_0 expressions derived from eco-epidemiological models of the transmission of *Trypanosoma cruzi*

A model for Chagas disease involving transmission by vectors and blood transfusion (Velasco-Hernandez, 1994).

Velasco-Hernandez 1994 proposed a model including blood transfusion and a distinction between two disease stages (acute and chronic).

$$R_0 = \frac{1}{2}\left(R_1 + \sqrt{{R_1}^2 - 4R_2}\right)$$

$$R_1 = \left(\frac{1}{\mu+\nu+\sigma}\right)\left(h_0 + \frac{h_1\sigma}{\mu}\right)\ ,\ R_2 = \left(\frac{L\alpha\mu}{\Lambda\delta^2(\mu+\nu+\sigma)}\right)\left(\beta_0 + \frac{\beta_1\sigma}{\mu}\right)$$

Where :

h_0 is the rate of infection through blood transfusion by hosts in acute stage.

h_1 is the rate of infection through blood transfusion by hosts in chronic stage.

Λ is the birth term of hosts.

L is the birth term of vectors.

μ is the host death rate.

$\delta$is the vector death rate.

σ^{-1} is the mean average time in acute phase.

$\nu = \nu_0 + \nu_1$ is the virulence (additional death rate) in stages acute and chronic.

$\alpha = ab$

$$\beta_i = ac_i$$

a is the number of bites of infected vectors per time unit.

b is the proportion of bites of infected vectors producing infection.

c_i is the proportion of bites on infected hosts producing infection in the vector for acute (0) and chronic (1) hosts.

A mathematical model for Chagas disease with infection-age-dependent infectivity (Inaba and Sekine, 2004).

Inaba & Sekine 2004 used a model including blood transfusion and a continuous infection-age τ – time elapsed since infection – as a substitute for the traditional compartments division between stages acute, chronic asymptomatic and chronic symptomatic.

$$R_0 = \frac{1}{2}\left(\langle h, \Gamma \rangle + \sqrt{\langle h, \Gamma \rangle + \frac{4\alpha b_2 \mu_1 \langle \beta, \Gamma \rangle}{b_1 \mu_2^2}} \right)$$

$h(\tau) = k\acute{h}(\tau)$

$\Gamma(\tau) = exp\left(-\mu_1 \tau - \int_0^{\tau} \gamma(\sigma) d\sigma\right)$: survival rate at duration τ in the infected stage.

$\beta(\tau) = a\acute{\beta}(\tau)$

With $\langle f, g \rangle = \int_0^{+\infty} f(\tau) g(\tau) d\tau$

$$\alpha = ac$$

a is the number of bites per vector per unit time.

c is the proportion of infected bites that give rise to infection.

k is the average number of blood transfusion per infected host per unit time.

$\acute{h}(\tau)$ is the probability that a blood transfusion from infected hosts with infection-age τ infects the susceptible host.

$\acute{\beta}(\tau)$ is the proportion of bites to infected hosts with infection-age τ that give rise to infection in vector.

$\gamma(\tau)$ is the rate of removal from infected status of infected hosts with infection-age τ.

b_1 is the hosts birth rate.

b_2 is the vectors birth rate.

μ_1 is the hosts death rate.

μ_2 is the vectors death rate.

The role of adaptations in two-strain competition for sylvatic Trypanosoma cruzi transmission (Kribs-Zaleta and Anuj Mubayi, 2012).

Kribs-Zaleta & Mubayi 2012 established a R_0 expression taking into account all the known transmission modes of *T. Cruzi* :

$$R_0 = \frac{1}{2}\left(p + \sqrt{p^2 + 4\frac{\tilde{\beta}_h \tilde{\beta}_v}{\mu_h \mu_v}} \right)$$

Where :

$$\tilde{\beta}_h = \beta_h min\left(\frac{Q}{Q_v}, 1\right) + \rho H min\left(\frac{Q}{Q_h}, 1\right)$$

$$\tilde{\beta}_v = \beta_v min\left(\frac{Q_v}{Q}, 1\right)$$

$$\tilde{\mu}_v = \mu_v + H min\left(\frac{Q}{Q_h}, 1\right)/Q$$

$Q$is the vector–host population density ratio.

H is the (maximum) per-host predation rate.

ρ is the estimated proportion of hosts infected after consuming an infected vector.

p is the vertical transmission probability.

Q_h is the threshold vector–host density ratio for predation.

Q_v is the threshold vector–host density ratio for bloodmeals.

μ_h and μ_v are the death rates of resp. the host and the vector.

$\beta_h \wedge \beta_v$ are the infection rates of resp. the host and the vector. Note that in Kribs-Zaleta & Mubayi 2012, $\beta_h = \beta_{hmax} \cdot x$, where $x$is the strain's adaptation to stercorarian transmission, and β_{hmax} is the maximum infection rate of the stercorarian transmission mode.

It must be noticed that this R_0 formula is reused (sometimes with variants) as a basis in other works of the same authors, such as :

The role of the ratio of vector and host densities in the evolution of transmission modes in vector-borne diseases. The example of sylvatic Trypanosoma cruzi. Perrine Pelosse, Christopher M. Kribs-Zaleta, Journal of Theoretical Biology 312 (2012) 133–142.

Graphical analysis of evolutionary trade-off in sylvatic Trypanosoma cruzi transmission modes, 2013, Kribs-Zaleta. Journal of Theoretical Biology 353 (2014) 34–43.

Vector Consumption and Contact Process Saturation in Sylvatic Transmission of T. cruzi, Christopher Kribs-Zaleta, Mathematical Population Studies, 13:135–152, 2006.

Household Model of Chagas Disease Vectors (Hemiptera: Reduviidae) Considering Domestic, Peridomestic, and Sylvatic Vector Populations (Stevens et al., 2013).

Stevens et al 2013 derived a particular expression of R_0 for a system where the unit of infection is not the infected individual but the dwellings inhabited by hosts, which include human houses, peridomestic structures (e.g., corrals), and sylvatic dwellings (e.g., animal nests, caves, and rodent burrows). It considers several environments (houses on one hand, and corral/sylvatic dwellings on the other hand) and includes the effect of insecticide treatment.

$$R_0 = \frac{1}{2}\left(\frac{(1-\eta_1)\beta_1 c_1 T_1}{a_1 b_1 N_1} + \frac{(1-\eta_2)\beta_2 c_2 T_2}{a_2 b_2 N_2} + \sqrt{\frac{4\eta_1 \beta_1 c_1 T_1 \eta_2 \beta_2 c_2 T_2}{a_1 b_1 N_1 a_2 b_2 N_2} + \left[\frac{(1-\eta_1)\beta_1 c_1 T_1}{a_1 b_1 N_1} + \frac{(1-\eta_2)\beta_2 c_2 T_2}{a_2 b_2 N_2}\right]^2}\right)$$

Environment types are identified by a number subscript : 1=houses, 2= corral/sylvatic.

η_i is the fraction of their life that vectors of environment$i$ spend in the other environment.

N_i is the total number of dwellings.

T_i is the number of insecticide-treated dwellings.

β_i is the infestation rate of dwellings.

$a_i = \mu_i + \gamma_i$,

$b_i = \mu_i + \gamma_i + \theta_i$,

$c_i = \mu_i + \gamma_i \sigma_i + \theta_i$,

where :

μ_i is the “mortality” rate of dwellings.

$\gamma_i$is the insecticide spraying rate.

$\theta_i$is the waning rate (represents the amount of time after application that the insecticide is effective).

$\sigma_i$is the spraying efficiency (proportion of dwellings that is insect-free after spraying).

A metapopulation model for sylvatic T. cruzi transmission with vector migration (Crawford and Kribs-Zaleta, 2014)

Crawford & Kribs-Zaleta 2014 derived a number of R_0 expression for a large variety of cases considering a diversity of hosts and vectors as well as a diversity of patches of population, with migration between patches. Here, we will not list the totality of R_0 expression they exposed in this paper, but we will reproduce one that considers two hosts, two vectors and three transmission cycles as an example of a multi-host *T. Cruzi* model.

$$R_0 = \sqrt{\frac{1}{2}\left(P + \sqrt{P^2 - 4Q}\right)}$$

$P = f_1 + f_2 + f_3$, Qf_1f_3 , $f_1 = \frac{(1-q_W)\beta_G \beta_{W2}}{\mu_G \mu_W}\frac{N_{G2}}{N_{W2}}$, $f_2 = \frac{q_W \beta_{SW} + (1-q_S)\beta_{WS}}{\mu_S \mu_W}\frac{N_{S2}}{N_{W2}}$, $f_3 = \frac{q_S \beta_R \beta_{S2}}{\mu_S \mu_R}\frac{N_{S2}}{N_{R2}}$

f1 represents the T. gerstaeckeri-woodrat transmission cycle.

f2 represents the T. sanguisuga-woodrat cycle.

f3 represents the T. sanguisuga-raccoon cycle.

Subscripts : S = T. sanguisuga, R = Raccoon, G = T. gerstaeckeri, W = Woodrat.

q_W is the proportion of Woodrats susceptibles who might be infected by infected$T. sanguisuga$; $1 - q_W$ is the proportion of those who might be infected by infected T. gerstaeckeri.

q_S is the proportion of T. sanguisuga susceptibleswho might be infected by infected raccoons; $1 - q_S$ is the proportion of those who might be infected by infected woodrats.

β_G is the transmission rate by infected T. gerstaeckeri.

β_R is the transmission rate by infected Raccoons.

β_{W2} is the transmission rate by infected Woodrats to T. gerstaeckeri.

β_{S2} is the transmission rate by infected T. sanguisuga to Raccoons.

β_{SW} is the transmission rate by infected T. sanguisuga to Woodrats.

β_{WS} is the transmission rate by infected Woodrats to T. sanguisuga.

N_{G2} , N_{S2} , N_{R2} , N_{W2} are asymptotic values for populations.

μ_i are death rates.

Broad patterns in domestic vector-borne Trypanosoma cruzi transmission dynamics (Peterson et al., 2015)

Peterson et al 2015 calculated a R_0expression including the effect of vector control, several disease stages (acute - a, chronic asymptomatic - i, chronic with determinate disease - d) with two hosts : humans and synanthropic animals.

$$R_0 = \sqrt{\left(\frac{\beta c_{vN} V}{(N+R)D\mu_b}\right)\left(\frac{\beta c_R c_{vR} p_v R}{\mu_R} + \frac{N}{\delta + \mu_N}\right)\left[\beta c_a + \frac{\beta c_i \delta}{\sigma + \mu_N} + \frac{\beta c_d \delta \sigma}{(\sigma + \mu_N)(\alpha + \mu_N)}\right]}$$

Where :

β is the triatomine-host contact rate.

c is the probability of infection of uninfected bugs from humans with status * : a/initial acute, i/indeterminate asymptomatic, d/determinate disease, or from animals (R).

$\overset{v\,*}{c}$ is the probability of infection from infected bugs for animals (*=R) and humans (*=N).

μ is the death rate for human hosts (*=N), animal hosts (*=R), and triatomine vectors (*=b).

α is the death rate in stage d.

δ is the rate of movement from acute stage to chronic asymptomatic stage.

σ is the rate of movement from asymptomatic stage to determinate disease stage.

R is the total number of synanthropic animals.

V is the total number of infected vectors.

Ecology, Evolution and Control of Chagas Disease: A Century of Neglected Modelling and a Promising Future (Nouvellet et al., 2015).

Nouvellet et al 2015 derived three expressions for R_0corresponding to three different saturation situations : a situation where the number of hosts is very high in regard of the number of vectors (and vectors can feed to satiety), a situation where the number vectors is too high (which leads to host irritability), and an intermediate situation where vectors must struggle to find hosts. The first formula among the three that follows corresponds to the high host density situation, the second one corresponds to the intermediate situation, and the third one corresponds to the high vector density situation :

$$R_0 = \sqrt{\frac{(b^{max})^2 p_{h,v} p_{v,h} \frac{N_v}{N_h}}{\mu_v \mu_h}}\ , R_0 = \sqrt{\frac{r^2 p_{h,v} p_{v,h} N_v N_h}{\mu_v \mu_h}}\ ,\ R_0 = \sqrt{\frac{(b^{max})^2 p_{h,v} p_{v,h} \frac{N_h}{N_v}}{\mu_v \mu_h}}$$

Where :

b^{max} represents the maximum number of vector bites.

N_h is the contact rate per host.

N_v is the contact rate per vector.

r is the finding rate.

$p_{h,v}$ is the per contact probability of pathogen transmission from vector to host.

$p_{v,h}$ is the per contact probability of pathogen transmission from host to vector.

References:

Crawford, B., & Kribs-Zaleta, C. (2014). A metapopulation model for sylvatic *T. cruzi* transmission with vector migration. *Mathematical Biosciences and Engineering: MBE*, *11*(3), 471–509.

Inaba, H., & Sekine, H. (2004). A mathematical model for Chagas disease with infection-age-dependent infectivity. *Mathematical Biosciences*, *190*(1), 39–69. https://doi.org/10.1016/j.mbs.2004.02.004

Kribs-Zaleta, C. M., & Mubayi, A. (2012). The role of adaptations in two-strain competition for sylvatic *Trypanosoma cruzi* transmission. *Journal of Biological Dynamics*, *6*(2), 813–835. https://doi.org/10.1080/17513758.2012.710339

Nouvellet, P., Cucunubá, Z. M., & Gourbière, S. (2015). Chapter Four - Ecology, Evolution and Control of Chagas Disease: A Century of Neglected Modelling and a Promising Future. In R. M. A. and M. G. Basáñez (Ed.), *Advances in Parasitology* (Vol. 87, pp. 135–191). Academic Press. Retrieved from http://www.sciencedirect.com/science/article/pii/S0065308X14000050

Peterson, J. K., Bartsch, S. M., Lee, B. Y., & Dobson, A. P. (2015). Broad patterns in domestic vector-borne *Trypanosoma cruzi* transmission dynamics: synanthropic animals and vector control. *Parasites & Vectors*, *8*. https://doi.org/10.1186/s13071-015-1146-1

Stevens, L., Rizzo, D. M., Lucero, D. E., & Pizarro, J. C. (2013). Household model of Chagas disease vectors (Hemiptera: Reduviidae) considering domestic, peridomestic, and sylvatic vector populations. *Journal of Medical Entomology*, *50*(4), 907–915.

Velascohernandez, J. X. (1994). A Model for Chagas Disease Involving Transmission by Vectors and Blood Transfusion. *Theoretical Population Biology*, *46*(1), 1–31. https://doi.org/10.1006/tpbi.1994.1017
